# Deep conservation of ribosome stall sites across RNA processing genes

**DOI:** 10.1093/nargab/lqab038

**Published:** 2021-05-25

**Authors:** Katarzyna Chyżyńska, Kornel Labun, Carl Jones, Sushma N Grellscheid, Eivind Valen

**Affiliations:** Computational Biology Unit, Department of Informatics, University of Bergen, Bergen 5020, Norway; Computational Biology Unit, Department of Informatics, University of Bergen, Bergen 5020, Norway; Computational Biology Unit, Department of Informatics, University of Bergen, Bergen 5020, Norway; Department of Biological Sciences, Durham University, DH1 3LE, UK; Computational Biology Unit, Department of Informatics, University of Bergen, Bergen 5020, Norway; Department of Biological Sciences, Durham University, DH1 3LE, UK; Department of Biological Sciences, University of Bergen, Bergen 5020, Norway; Computational Biology Unit, Department of Informatics, University of Bergen, Bergen 5020, Norway; Sars International Centre for Marine Molecular Biology, University of Bergen, Bergen 5008, Norway

## Abstract

The rate of translation can vary depending on the mRNA template. During the elongation phase the ribosome can transiently pause or permanently stall. A pause can provide the nascent protein with the time to fold or be transported, while stalling can serve as quality control and trigger degradation of aberrant mRNA and peptide. Ribosome profiling has allowed for the genome-wide detection of such pauses and stalls, but due to library-specific biases, these predictions are often unreliable. Here, we take advantage of the deep conservation of protein synthesis machinery, hypothesizing that similar conservation could exist for functionally important locations of ribosome slowdown, here collectively called stall sites. We analyze multiple ribosome profiling datasets from phylogenetically diverse eukaryotes: yeast, fruit fly, zebrafish, mouse and human to identify conserved stall sites. We find thousands of stall sites across multiple species, with the enrichment of proline, glycine and negatively charged amino acids around conserved stalling. Many of the sites are found in RNA processing genes, suggesting that stalling might have a conserved role in RNA metabolism. In summary, our results provide a rich resource for the study of conserved stalling and indicate possible roles of stalling in gene regulation.

## INTRODUCTION

Besides encoding the amino acid sequence of a protein, the coding region of mRNA can contain secondary signals affecting the regulation of the gene. These regulatory signals can modulate elongation rates, leading to translation bursts and pauses and determine how efficiently proteins are synthesized (1,2). While many of these signals are likely tuning the rate of synthesis in a subtle fashion, some signals have been shown to cause longer-lasting pauses or ’stalls’.

These stalls can have important biological consequences allowing time for the recruitment of various machinery to facilitate subsequent processes, such as membrane targeting or co-translational protein folding (2–4). For instance, pausing upon the emergence of the signal peptide from the ribosome exit tunnel promotes recruitment of the signal recognition particle and subsequent targeting of secretory proteins to the endoplasmic reticulum (5). Slowing down translation downstream of protein structural domains would in turn allow time for the domains to fold into lower-energy folding intermediates (2,6). However, if the ribosome stalls due to aberrant translation, it may trigger recruitment of ribosome-associated protein quality control machinery to degrade the nascent peptide through pathways such as nonsense-mediated decay (NMD) or no-go decay (NGD) (3,7–9).

Several causes for stalling have been suggested, such as (i) specific amino acids (e.g. proline) in the P- and A-site attenuating the rates of peptide bond formation (10,11), (ii) positively charged residues (12) or non-optimal codon clusters in the nascent peptide, interacting with ribosome exit tunnel (5,13) or (iii) mRNA secondary structure blocking progression of translating ribosomes (14,15). However, it is unclear how widespread each of these causes are and to what extent these are functional.

Ribosome profiling, the sequencing-based transcriptome-wide capture of ribosomal occupancy, can offer unique insight into the dynamics of ribosome translocation based on the distribution of sequencing reads from ribosome protected fragments. Indeed, previous analyses of ribosome profiling data have revealed widespread presence of sites with a high abundance of reads, assumed to be strong ribosomal pauses (16). However, past studies have also demonstrated a wide range of biases in ribosome profiling data as well as high local variations between data from individual libraries and experiments (10,17–19). Analysis of a single library or experiment is therefore particularly vulnerable to biased results which again can confound the search for regulatory mechanisms.

The deep conservation of the translation machinery suggests that many functionally important stall sites may also be conserved. Here, we explore these by collecting 20 publicly available ribosome profiling datasets from five phylogenetically diverse model organisms in order to identify stall sites conserved across multiple species and experiments. By their conservation, these stalls are likely to represent functional sites. We characterize their biological contexts and uncover signs of conserved mechanisms as well as a conserved role for stalling in RNA processing.

## MATERIALS AND METHODS

### Ribosome profiling data

The raw sequencing data were downloaded from the Sequence Read Archive (SRA). The normal condition libraries include yeast: SRR387905 (20), SRR1520317 cycloheximide (CHX)-treated and SRR1520325 (no drug) (18); fruit fly S2 cells: SRR942880 (21), SRR6930625 (22) and SRR3031135; zebrafish embryos at different stages of development: 2 h post fertilization (hpf) - SRX399824, SRX399826, SRX399828 (23), SRR5893147, SRR5893148 (24) and SRR1039873 (25), 4 hpf - SRR836195 (26) and SRR1039876 (25), 6 hpf - SRR836196 (26) and SRR1039879 (25); mouse: embryonic stem cells - SRR315601, SRR315602 (CHX-treated), SRR315616, SRR315617, SRR315618, SRR315619 (no drug) (16) and 3T3 fibroblasts - SRR1039863 (25); human: fibroblasts - SRR609197 (CHX-treated) and SRR592961 (no drug) (27), mitotic HeLa cells - SRR970587 (28) and HEK293 cells - SRR1039861 (25). Overview of data libraries is shown in Supplementary Table S1.

### Ribosome profiling data processing

Each of the raw ribosome profiling sequencing libraries was processed in the following way: (i) trimming adapters specific for each experiment and low quality bases with cutadapt (29), keeping reads of minimum length of 20 nucleotides; (ii) removing reads mapping to ribosomal RNAs from SILVA rRNA database (30) and organism-specific non coding RNAs from Ensembl (31) using Bowtie2 (32); (iii) aligning the remaining reads to organism-specific reference transcriptome with TopHat2 (33), allowing up to two mismatches. The Ensembl genome versions used were R64-1-1 for yeast, BDGP6 for fruit fly, GRCz10 for zebrafish, GRCm38 for mouse and GRCh38 for human; (iv) selecting the periodic footprint lengths and assigning them to P-site nucleotides with Shoelaces (34). The selected lengths and offsets are shown in Supplementary Table S1. The ribosome meta-profiles (Supplementary Figure S1) were plotted by taking the last 30 nucleotides of 5’UTR, first and last 60 nucleotides of CDS and 30 first nucleotides of 3’UTR (from the genes that contain features of minimum those lengths) and superimposing them, taking into account the length of the footprint.

### Stall site calling

Ribosome footprint profiles for each transcript were constructed by quantifying the number of footprints assigned to each nucleotide position. For organisms with multiple transcripts per gene (all but yeast), only the longest transcripts were used. For prediction of stall sites, a further subset of well-expressed transcripts, defined as having a median codon coverage higher than zero, were selected. As there are usually high peaks over start and stop codons due to prolonged time of initiation and elongation, the first and the two last codons of each CDS (start, stop and a codon before stop codon) were excluded from further analysis to avoid skewing the footprint distribution over transcripts. The codon coverage per transcript was then transformed into *z*-scores, and the stall sites were identified as codons with coverage higher than a certain threshold (experimental cut-off of 5.0 was chosen). The peaks within the first five codons of CDSs were excluded to avoid these caused by accumulation of ribosomes at the beginning of CDSs in some libraries. To further increase the confidence that defined peaks are indeed stall sites and not experimental or sequencing biases, the peak had to occur in at least two different organisms (see below) to be considered a stall site. The number of peaks in each library and overlap between libraries is shown in Supplementary Table S2.

### Conservation analysis

Sets of homologous genes for each organism were retrieved from Ensembl using Biomart (35) querying. The transcript sequences for each set of homologs were aligned together using Clustal Omega (36) version 1.2.1. To alleviate potential alignment issues, such as extensive differences between sequences of one or more homologs that could lead to large gaps in alignments and possible local misalignment, we re-aligned the sequences iteratively. We started with the alignment of the maximum set of five homologs, when available. The positions of stall sites for each organism were then cross-checked among homologous transcripts to account for insertions and deletions. If the stall site occurred at the same or adjacent codon (3 nucleotides upstream or downstream of the peak to account for possible minor differences in P-site footprint assignment) in homologs in given organisms, it was considered to be conserved in these. If it did not, we continued aligning all subsets of homologs from 4 different organisms, then 3, and finally 2, until all putative stall sites were accounted for. The list of conserved stall sites is presented in the Supplementary Table S3. Note that Xbp1 is not listed in the results table, as it has been generated based on alignments of longest transcript per gene from Ensembl annotations. Manual inspection revealed that the correct transcripts of Xbp1s/u for mouse and humans are missing in Ensembl, which shifted the alignments. Therefore these had to be downloaded from RefSeq (37), and the shorter Xbp1u transcripts have been aligned and analyzed as others. This was the only case of discrepancy between Ensembl and RefSeq annotations in the analyzed genes. The likelihood of finding conserved stalling by random was calculated with binomial coefficients. For two organisms:

$$\begin{eqnarray*} \mathrm{p} = \frac{{n\atopwithdelims ()k} - {n-m\atopwithdelims ()k}}{{n\atopwithdelims ()k}} = \frac{(\frac{n!}{k!(n-k)!} - \frac{(n-m)!}{k!(n-m-k)!})}{\frac{n!}{k!(n-k)!}} \end{eqnarray*}$$

where *n* = 500 (a median gene length), *k* = 3 (average number of peaks per gene) and *m* = 3 (number of potential codons matching a peak, as we consider adjacent codons as well as the peak codon), yielding *P* = 0.018. For three organisms, *P* = 0.018*0.018, etc. There are 4263 homologs being compared between mouse and human, yielding 0.018*4263 = 230 pauses to be found at the same position by random. Adding zebrafish, the probability drops to ∼2.6 pauses found by random, adding the fourth and fifth organisms probability drops to a fraction of a pause. To test for significance, we performed a one-tailed binomial test with H0: *P* < 0.018 and H1: *P* > 0.018 and 95% confidence interval. With *P*-value <2.2e-16, we accept the null hypothesis that the true probability of finding conserved stalling is <0.018.

### SNP analysis

For frequent to rare substitution analysis, *de novo* SNPs in the H2 library (which has the highest coverage among the human libraries) were called with bcftools (38) with default settings, predicting 14 SNPs with low coverage. None of them were associated with CSSs. Human CSSs were then checked for association with all known single-nucleotide polymorphisms (SNPs) from dbSNP database (39), clinically associated SNPs from ClinVar database (40), and also the 14 novel SNPs from the H2 library. For each stall site, a random codon on the same gene has been used as control. We estimated that the chance of finding a SNP at a specific position in the CDS regions (see Stall site calling) is 0.009. Therefore, the expected count of SNPs overlapping with control stall sites or conserved stall sites is 65 (dbSNP, ClinVar). In the H2 library, we find 55 (dbSNP) and 54 (ClinVar) SNPs that overlap with control stall sites, and 54 (dbSNP) and 53 (ClinVar) that overlap with conserved stall sites. Restriction to only non-synonymous SNPs results in 27 (dbSNP) and 31 (ClinVar) over control stall sites, while 35 (dbSNP) and 33 (ClinVar) over conserved stall sites.

### Sequence analysis

For amino acid heatmaps, we calculated the frequency of occurrence of specific amino acids and their 2-mers and 3-mers in the positions of 10 amino acids upstream and downstream of CSSs. For nascent peptide analysis, the 30 amino acids upstream of CSSs that would span the ribosome exit tunnel were summed up depending on properties (positively charged, negatively charged, special). For control, we sampled 10 000 times a random position from each of the transcripts with CSSs and calculated the average. Sequence logos were created with WebLogo3 (41) for all CSSs, as well as split by the most common amino acids. Additionally, we performed motif discovery with MEME suite (42) but found no reliable result. To test for statistical significance of the [CGA][CGA]N motif, we randomly sampled 11 codons for every stall site from corresponding gene sequences, repeated 1000 times and calculated the average number of motif matches. We found 989 motifs on average, compared to 1419 matches for CSSs (out of 2397 analyzed sequences). We performed a Pearson’s Chi-squared test with 1 degree of freedom. We found that the motif is statistically significant, with a *P*-value < 2.2e-16. For analysis of arrest peptide sequences, we calculated amino acid frequencies in the 40 amino acids upstream of CSSs, control sites (randomly chosen on the same genes, non-overlapping with CSSs regions) and all human protein sequences. There was no significant difference in frequencies of any of these (per 1 kilobase) compared to controls (chi-square goodness of fit test with significance level of 0.05, *P*-value of 1 in both cases).

### Structure analysis

*In silico* mRNA secondary structures of transcripts were predicted by calculating a minimum free energy (MFE) in a 51-nucleotide sliding window over CDSs using RNAfold program (43), as done previously (15,44). For structure analysis, we used only stall sites positioned >30 nucleotides downstream from the start codon and 60 nucleotides upstream from the stop codon to avoid the decreased structure at the beginning and end of CDSs impacting the results of the analysis. This left 627 CSSs which were not explained by sequence features. As control, for each stall site we picked at random a position in the given transcript that neither overlaps with the region around the stall site (-30/+60 bases) nor the regions around start/stop codon as mentioned above. This was repeated 10 000 times with different random positions and averaged. We overimposed averaged regions around CSSs and average control in a meta plot. Additionally, we included a control preserving the amino acid content around the CSSs. This was done by taking the exact same region around the CSSs, with the stalling codon kept in place, but with the codons in positions -30:-1 and 1:60 permuted 100 times. For these permuted regions we calculated the MFE as above, and averaged over all repetitions.

### Gene Ontology enrichment

The genes with conserved stall sites present in human and at least one other organism were subject to Gene Ontology enrichment analysis. The enrichment was performed with clusterProfileR package (45) against a background of well-expressed homologs, as the set of stall sites was biased towards well-expressed homologs as well. As controls, we used well-expressed homologs without peaks (maximum *z*-score<5) and well-expressed homologs with non-conserved peaks (see Supplementary Tables S2 and S6). GO analyses for both controls returned no results. To test for biases in the CSS-containing and control groups we compared mean ribosome coverage and amino acid distribution in the three sets (see Supplementary Figure S11A and S11F). There were significant differences in some of the human libraries (two-sample Mann–Whitney *U* test). To control for these differences, we sampled from the well-expressed transcripts not containing CSSs groups of similar size, ribosome coverage (Supplementary Figure S11B) and translational efficiency (Supplementary Figure S11C) to the CSS-containing sample. Translational efficiency was calculated as FPKM (fragments per kilobase of transcript per million mapped reads) of the H2 sample divided by the FPKM of the corresponding RNA-seq sample (SRR592966 (27)). Lack of significant difference between the controls and CSS group were ascertained with two-sample Mann–Whitney *U* test (*P*-values close to 1). Similarly, we compared amino acid distributions in the sets (Supplementary Figure S11D). There was no significant difference in amino acid distribution of the CSS-containing and control groups (per 1 kilobase) compared to the background of the whole genome or well-expressed proteins only (chi-square goodness of fit test with significance level of 0.05, *P*-value was 1 in all cases). Finally, to account for minor differences in alignment score and peak number between CSSs and well-expressed homologs with non-conserved peaks, we subset the non-conserved peak set to match alignment score and peak distributions (Supplementary Figure S11D and S11E, *P*-values close to 1 with two-sample Mann–Whitney *U* test). The alignment score was calculated as Levenstein distance between two aligned sequences (human and another organism, as in CSSs). GO analyses on these returned no enriched terms.

### Exon and protein domain boundary analysis

For splicing factors binding sites, we analyzed 200 nucleotides flanking CSSs for 6-mer content. Given the annotated splice junctions of the transcripts, we calculated the distance from CSSs in nucleotides to the upstream and downstream exon boundaries, and relative position of CSSs within the exons, excluding the first 45 and last 3 nucleotides. For protein domain boundary analysis, we downloaded protein domain annotations from CATH database (46). We analyzed 1317 CSSs on 1100 genes that had an annotated domain, calculating the minimum distance to the upstream C-terminal protein domain boundary. For control, we used the random positions on the same genes. To predict disorder in CSS-containing proteins, we used DisEMBL (47). We extracted disorder scores for prediction of loops/coils around CSSs and averaged in a meta-plot. For control, we performed random selection of amino acids on the same proteins, excluding first 15, last 2 and those corresponding to CSSs, repeating 100 times for every gene. Additionally, we calculated the percentage of CSSs found in coils, as defined by default threshold of 0.516 versus average percentage of random sites. We performed a two-sample *t*-test for scores at CSSs vs random positions, yielding the difference statistically significant with *P*-value<2.2e-16.

### Transmembrane domain analysis

We downloaded all 1512 TM type I and 464 type II proteins available in UniProt (48) for human (annotation SL-9905 and SL-9906). Out of these, we selected those containing CSSs, resulting in 76 and 36 for type I and type II, respectively. We looked at the distribution of these in the genes, and found no overrepresentation at any certain position downstream of the signal peptide.

### Premature termination analysis

For the human libraries, we analyzed transcripts that contained CSSs and had at least 15 codons before and after the CSS contained within the body of the gene (excluding first 15 and last 2 codons). To check for reduction of signal 3’ of the pause site, we calculated log2 ratio of the mean ribosome coverage in the upstream region to the mean coverage in the downstream region from the CSS. For fragment length distribution, for libraries H1-H4 we calculated background distributions of all footprints. We extracted coverage 15 nt upstream and 15 nt downstream around all stop codons (for the transcripts that contain 3’UTRs) and created metaplots of average footprint lengths at these positions. Similarly, for each of the libraries, we extracted coverage around CSSs present in a given library and created metaplots as in the case of stop codons.

## RESULTS AND DISCUSSION

To identify stall sites that are conserved across species and that are robust to library preparation, we collected publically available ribosome profiling libraries from yeast (*Saccharomyces cerevisiae*), fruit fly (*Drosophila melanogaster*), zebrafish (*Danio rerio*), mouse (*Mus musculus*) and human (*Homo sapiens*). The choice of libraries was dictated by availability, sufficient level of coverage, and different experimental conditions, to be able to eliminate noise originating from particular experimental protocol or sequencing bias. All of the data are from wild type and unstressed cells.

**Figure 1.**
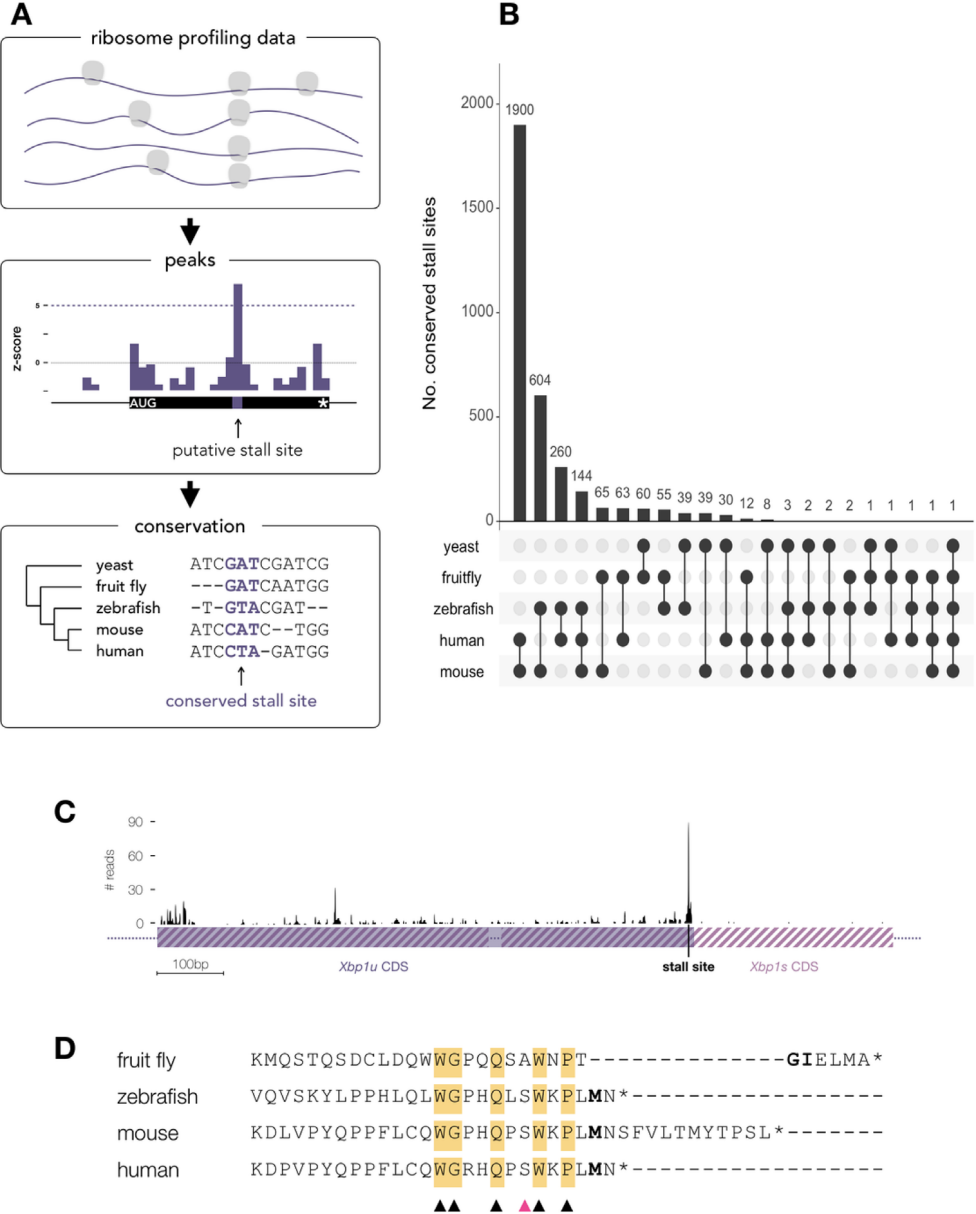
Conservation of stall sites across divergent eukaryotes. (**A**) Schematic representation of stall site analysis. (**B**) Number of conserved stall sites in homologous genes in yeast, fruit fly, zebrafish, mouse and human. Some stall sites are common in lower and higher vertebrates, indicating their importance in translation regulation. (**C**) Ribosome profile on the Xbp1 mRNA (here values for H3 library). The schematic of two isoforms is shown, the unspliced, shorter Xbp1u (solid purple) and spliced, with 3’ extension, Xbp1s (striped purple). The stall site at the 3’end of Xbp1u is indicated. (**D**) Alignment of C-terminal peptide sequences of Xbp1u from fruit fly, zebrafish, mouse and human. The amino acids in the P-site position where the stalling occurs are indicated in bold. Conserved amino acids are highlighted in yellow, while the ones which are most likely critical for stalling are indicated with triangles. A pink triangle indicates a position where S to A mutation has been demonstrated to increase the translational pausing (50).

After mapping the ribosomal footprints to the transcriptome (see Materials and Methods section) we identified, for each ribosome protected fragment, the codon currently under translation (P-site). Importantly, this is a library-specific process and requires offsetting each fragment length by a specific amount calculated using Shoelaces (34) (Supplementary Table S1). This processing resulted in codon-resolution data as revealed by ribosome meta profiles (Figure 1A and Supplementary Figure S1), showing clear peaks over the first and/or last translating codons, increased coverage within the coding region (CDS), and in most cases three-nucleotide periodicity, as expected of a correctly P-shifted data (34,49).

### CHX causes initiation bias, while flash-freezing captures terminating ribosomes

The use of translational inhibitors has been shown to cause abnormalities in ribosome profiles. Specifically, in cycloheximide-treated samples, the slow diffusion of the drug allows translation to continue for a few codons before the CHX reaches 100% efficiency. This can be observed as an artifactual ’ramp’ at the 5’ end of the coding sequences (18,19). We observe the accumulation of initiating ribosomes in CHX-treated samples (Y1, Y2, Z3, Z5, Z7, M1, M3, H1, H3, H4), as well as those treated with emetine and rapamycin (F1, F2, F3). Interestingly, in samples where no drug was used or they have been flash-frozen before additional treatment with CHX (Y3, Z1, Z2, Z4, Z6, M2, H2), we observe minimal accumulation of ribosomes at the start codon; they however accumulate at the last sense codon of the CDS. This peak before stop codon likely comes from termination pausing, which allows time for the termination complex to assemble, release the peptide and dissociate. This termination peak is lost in CHX treated samples, due to the long time in the translational timescale required for CHX to reach equilibrium (18) which is likely too long to capture terminating ribosomes.

### Detection of robust and conserved stall sites to control for library bias

To obtain high-confidence stall sites (Figure 1A), we devised a method to detect stalls above the noise level and required these sites to be detected in at least two libraries from different organisms (see Materials and Methods section). As most ribosome profiling experiments have used CHX to ’freeze’ elongating ribosomes, this might skew the distribution of stall sites towards longer-lasting pauses. While flash-frozen samples may provide a more accurate picture of short-lived pauses, there is currently not enough data of this type to detect transient pausing above noise levels. We, therefore, limited our analysis to long-lasting pauses (1–2 s, given a mean decoding rate of 5.6 codons per second (16) and a slow inhibition by CHX allowing time for ribosomes to run-off for several codons (19)). Detection of such pauses should therefore be independent of the use of translation inhibitors and, given the support of multiple libraries, can be separated from artificial peaks produced by library-specific biases. Using this strategy we identified thousands of peaks in each of the libraries (see Supplementary Table S2), with only a small fraction of them replicated across experiments (yeast: 781; fruit fly: 1096; zebrafish: 167; mouse: 577; human: 674).

Stall sites that are evolutionary conserved are likely to have biological significance. We, therefore, compared the positions of peaks in homologous genes across all five analyzed organisms (see Materials and Methods section). This analysis revealed 3293 stall sites conserved in at least two organisms (Figure 1B and Supplementary Table S3). For human, we detected 2426 peaks in 1729 genes that are present in at least one other organism, and we will refer to these as ’conserved stall sites’ (CSS). The highest degree of conservation is unsurprisingly seen over the shortest evolutionary distance, between mouse and human homologs. These sites account for ∼10% and ∼18% of total peaks for mouse and human, respectively. Surprisingly, we find some pause sites conserved even in yeast, which is evolutionarily distant from the other organisms. For the remaining organisms the number of stall sites that are conserved only accounts for 1-4% of all peaks in all genes (Supplementary Table S2). To estimate the probability of this occurring by chance, we observed that well-expressed homologs have a median length of around 500 codons and contain an average of ∼3 peaks per gene. Under these parameters, the probability of finding peaks at the same position is 0.018 for two homologs, 3.2e-04 for three, 5.8e-06 for four and 1e-07 for five (see Materials and Methods section). Therefore, in our largest conserved group, mouse and human, we would expect 230 peaks to be found by chance (11% of the 2069 peaks that we identify). When expanding to zebrafish, we would expect <3 of the 178 sites to be found in the three organisms by chance. For four or five homologs less than one conserved peak is expected. The high degree of conservation of stall sites therefore supports the idea that conserved stalling is a non-random event (*P* < 2.2e-16, see Materials and Methods section) and that the sites that we identify are likely to have biological significance.

Stalling has been predicted from ribosome profiling previously (51). While most of the studies focused on bacteria (for a recent review on bacterial stalling, see (52)) or yeast, to date only Ingolia *et al.* 2011 looked at genome-wide stalling in higher eukaryotes (mouse) (16). Importantly, the pause sites in this and other studies are generally identified by the peak in read density alone and often in a single library. The seminal study of Ingolia *et al.*, 2011 reported 1500 strong ribosomal pauses, evolutionarily conserved and estimated to last for several seconds. Since pauses of this duration should be captured independently of the method used for translation inhibition, we compared the peaks derived from this study (M2) that did not use translation inhibitors with CHX-treated libraries from the same study (M1) using our peak calling strategy. With our method we recovered all 1500 peaks reported previously together with 16474 of novel sites in M2. However, when comparing these with the CHX-treated library M1, the overlap is only 308 sites that are present in both libraries. To further investigate whether the subset of stall sites repeated in both libraries was in agreement with the sequence motif reported to cause stalling (16), we analyzed the peptide sequence around these stall sites. As our methodology for calling peaks is largely similar to Ingolia et al. 2011 (see Materials and Methods section), the peaks found in library M2 were consistent with the previous report with a strong enrichment of glutamate or aspartate in the A-site preceded by a proline or glycine and then another proline (Supplementary Figure S2A). However, identical analysis for pauses present in the overlap of both M1 and M2 libraries revealed that the P-site is most likely to contain an aspartate or glycine (Supplementary Figure S2B). The bias was similar for the A-site, though less pronounced. Both stall sites also revealed an influence from double prolines, which has previously been shown to cause stalling (10). The low fraction of pause sites that is consistent across treatments and a changed sequence motif suggest that a significant number of sites reported previously are likely due to library bias and do not carry biological significance, though some could occur due to shorter transient pausing.

A well-characterized example of stalling within a eukaryotic CDS is the transcription factor XBP1. During impairment of protein folding in the endoplasmic reticulum (ER), commonly known as ER stress, the nascent chain of XBP1u (the shorter, unspliced isoform of the transcript) localizes to the ER membrane and stalls. While stalled, the spliceosome on this membrane cuts out a fragment of yet untranslated mRNA, changing the open reading frame of the transcript and as a result, produces an extended protein (50). Mutational and evolutionary analysis of XBP1u peptide revealed peptide module at the carboxyl terminus required for pausing and splicing, of which 15 amino acids were conserved in human, mouse, chicken, frog, and zebrafish and deemed necessary for stalling (50). The exact position of the stall site has been identified in mouse ribosome profiling data as a high peak over Asn256 codon in the A-site (16), corresponding to Met255 in the P-site. In our analysis, we identified peaks close to the 3’-end of the Xbp1u mRNA in all of the libraries in the organisms where the two isoforms exist (fruit fly, zebrafish, mouse and human; Figure 1C and Supplementary Figure S3). The position of the peak is at the Met in P-site, with Asn in the A-site in zebrafish, mouse and human. Interestingly, in fruit fly the identity of the P- and A-site codons is different, yet the peak occurs at the same position, as revealed by multiple sequence alignment (Figure 1D). In our analysis, the addition of fruit fly narrows down the number of conserved residues in the nascent peptide from 15 found before (50) to 5 amino acids, Pro in position -2 (relative to P-site at 0), Trp at -4, Glu at -7, Gly at -10 and Trp at -11. At position -5, the substitution of Ala (as in fruit fly) for Ser (as in the remaining organisms) has been shown to augment pausing (50). Therefore, we conclude that these six residues are most likely critical for stalling on Xbp1u.

### Proline, glycine and negatively charged amino acids are conserved mechanisms of stalling

**Figure 2.**
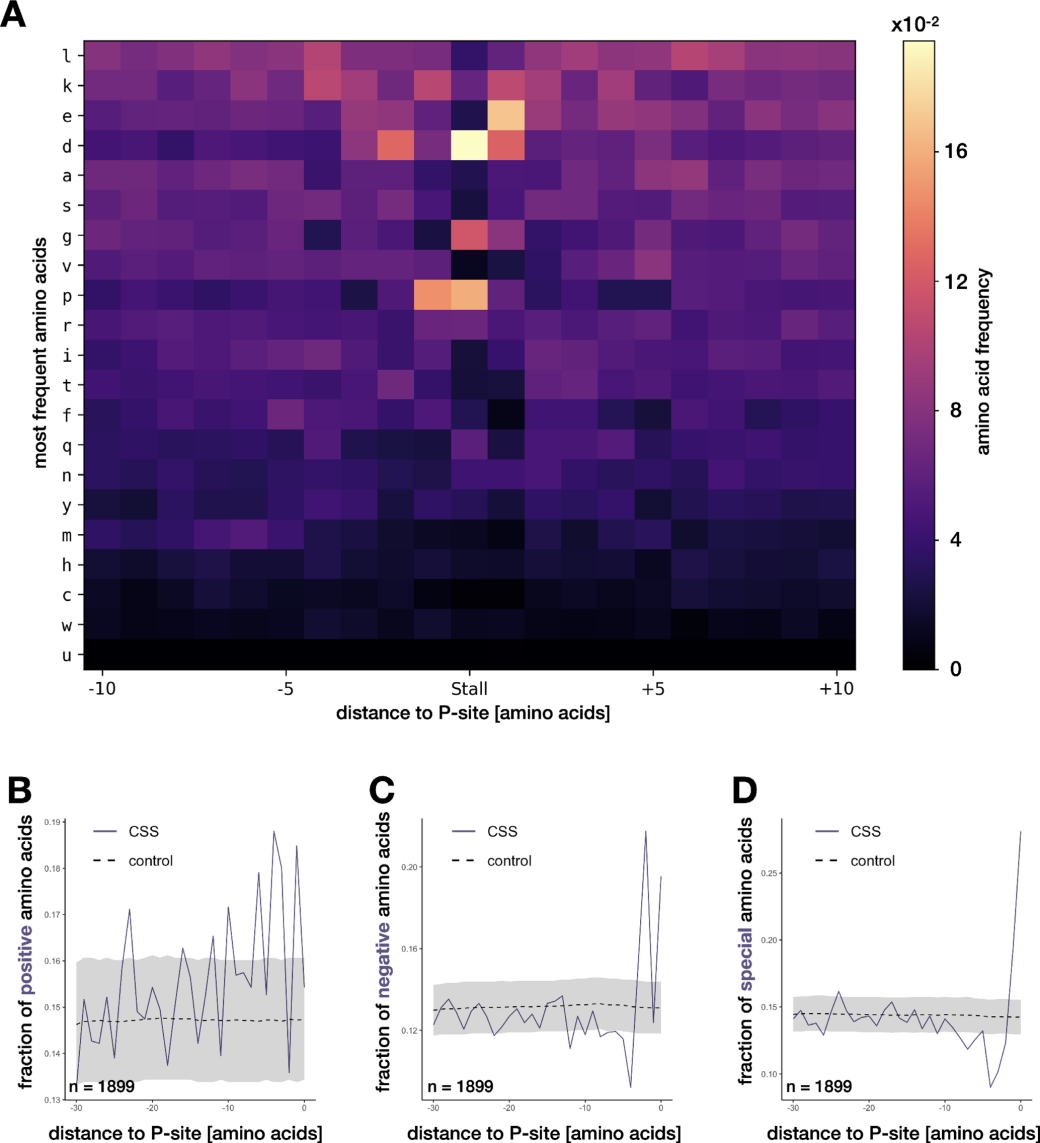
Mechanisms of conserved stalling. (**A**) Overrepresentation of amino acids around CSSs, sorted from most to least frequent, top to bottom. (**B**) Fraction of positively charged amino acids in the 30 amino acids upstream of the CSSs at P-site (0), spanning the exit tunnel versus background (shaded regions show background distribution, from 5th to 95th percentile). Similarly, (**C**) fraction of negatively and (**D**) special (proline and glycine) amino acids.

We investigated whether the organism-specific stall sites as well as CSSs are associated with factors that have been previously implicated in stalling by analyzing sequence and structure patterns around stall sites (see Materials and Methods section). We corroborate the results that found proline as a major contributor to stalling (10). Single proline amino acid in the P-site seems to indeed be one of the most influential individual contributors to conserved stalling, accounting for around 15% of CSSs (compared to around 6% of all codons in human coding for proline). The other significant contributors at P-site seem to be glycine (present in 12% CSSs, 7% in background) and aspartic acid (in 17% CSSs, 5% in background). Influential is also glutamic acid at A-site, found in 17% CSSs versus 7% in background, of which 10% do not overlap with Pro/Gly/Asp in P-site (Figure 2A). Interestingly, a recent study by Mohammad *et al.* (53) proposed improvements to bacterial ribosome profiling protocol (suggesting e.g. flash freezing) to remove confounding artifacts and improve resolution. Using the revised method, they obtained similar results to our analysis, with Pro/Gly/Asp as the main pausing contributors in bacteria. Similar analyses of subsets of CSSs conserved in three or more organisms, and for each organism separately returned similar enrichment of Pro/Gly/Asp (Supplementary Figure S13). The only exception to this was fruit fly, which lacked the enrichment of proline. A potential cause of this could be the lower conservation of the fruit fly nucleotide sequences, as compared to between human, mouse and zebrafish, which could result in different amino acid profiles. Alternatively, the different treatment for the fruit fly libraries (emetine and DMSO, see Supplementary Table S1) could potentially lead to differences in the ribosome profiling data.

We next asked whether similar biases could be observed in the nucleotide sequence by making a consensus logo of all CSSs (Supplementary Figure S4A). This revealed a bias towards a [CGA][CGA]N motif (*P*-value < 2.2e-16, see Materials and Methods section), which can be attributed to the codons for the enriched amino acids (proline: CCN, glycine: GGN, aspartic acid: GA[UC] at the P-site and glutamic acid: GA[AG] at the A-site). To investigate whether we could find any additional biases, we made individual logos for each of these amino acids (Supplementary Figure S4B). Together, 54% of all CSSs can be attributed to amino acid sequence, while the remaining 46% do not form any significant motif, neither in sequence logo nor by motif discovery (MEME) analysis (42). Importantly, the sequence logos split by amino acids revealed nucleotide context in codons at positions -3 to +1. Such bias is not found when looking at other Pro, Gly, Asp and Glu amino acids from CSS-containing genes that are not stall sites (not shown). To see if this sequence context can be explained by stretches of particular residues, we searched for amino acid 2-mers and 3-mers (Supplementary Figure S5). We do find significant stalling on proline, especially when present in doublets or triplets with another amino acid (XPP/PPX) (54). These and other k-mers can explain up to 5% of CSSs, however, they occur at positions -1 to +1, which does not account for bias at position -3 to -2. An explanation for the latter could be that this sequence context is recognized by the ribosome, in a mechanism similar to recognition of Kozak sequence at the translation initiation sites (55), as the two contexts are of similar intensity.

A controversial question has been whether the charge of amino acids plays a role in stalling. While some studies have claimed that newly synthesized, positively charged amino acids contribute to stalling (12), others have disputed this (10) or only found a subtle effect (56) and only in the absence of translation inhibitors (57). Others again found negatively charged amino acids contributing to stalling under certain conditions (11). Given the conservation of the translational machinery, it is likely that a charge-dependent mechanism will also be conserved between multiple organisms. We therefore analyzed the 30 amino acids upstream of stall sites that would span the ribosome exit tunnel. Using random sites in the same gene as a control we found a small contribution from positively charged amino acids present immediately upstream of the ribosome active site, particularly at positions -1 (E-site), -3, and -4. The proportion of CSSs that have these sequences however is small (2.3% of stalling cases) (Figure 2B) and can be fully attributed to consecutive lysine codons, which are present in nearly 3% of sequences just upstream of CSSs. Successive lysine codons are known to cause stalling by ribosome sliding, which have been demonstrated both in bacteria and eukaryotes (58–60), and not by their positive charge. Consecutive lysine residues are indeed the most frequent 2-mers and 3-mers that influence stalling (see Supplementary Figure S5A,B). Lack of enrichment of positively charged amino acids around CSSs could be a consequence of our stall site definition which detects stops at a single codon, since stalling caused by positive charge has been shown to be less defined, affecting average ribosome density over a larger region (12,56,57).

Interestingly, we find a much stronger contribution from negatively charged amino acids at position -2 (first amino acid in the exit tunnel), see Figure 2C. Most of them co-occur with the four amino acids found to be the strongest stalling contributors, especially Gly (34% of Gly-related CSSs; for others, the percentages are Asp: 22%, Pro: 20%, Glu: 14% and the rest: 15%). Altogether, in total, 12% of all CSSs are Gly/Pro/Asp/Glu-associated and have a negatively charged amino acid at position -2, additional 7% have the charge at -2, but do not have these amino acids. As the entrance to the exit tunnel is narrow (61), we hypothesize that upon encountering it, the negative charge of amino acids might repel the negative charge of the exit tunnel and slow down translation. Consistent with this, it was previously shown that the stalling on bacterial MifM depended on negative charges residing proximally to the multiple arrest points (62), possibly due to negative charge-mediated inhibitory interaction with the ribosome tunnel. Similarly, another study comparing multiple eukaryotic organisms, found that in some of the datasets, negative charge of amino acids can induce stalling (11). However, how this happens mechanistically, is not currently known. Interestingly, the negatively charged amino acids at position -2 and positively charged at -1, -3 and -4 are never found together (Figure 2B and C). Finally, as already observed, we see a large contribution from the ’special’ amino acids: proline and glycine, at the P-site, that are found in 27% CSSs (Figure 2D). Similar analyses of subsets of CSSs conserved in three or more organisms, and for yeast, fruit fly, zebrafish and mouse are shown in Supplementary Figure S14. These show similar patterns with the exception of fruit fly and yeast that lack the enrichment of negatively charged amino acids at position -2. Lack of these in fruit fly could be due to the differences in library treatment (Supplementary Table S1) or lower conservation. Alternatively, both yeast and fruit fly have a low number of CSSs, which could simply be insufficient to observe an enrichment.

Additionally, it has been shown that some specific sequences of amino acids in nascent peptides enhance ribosome arrest in bacteria (13,63). As these are often aromatic (Phe, Trp, Tyr) and usually located at different positions in different peptides, we analyzed the frequencies of amino acids in the region upstream of CSSs spanning the ribosome exit tunnel. However, we found no significant difference in total frequencies of any amino acid or group of amino acids compared to controls (Supplementary Figure S12).

Overall, we can explain 63% of CSSs by sequence features: 44% by amino acids at P-site (15% Pro, 12% Gly and 17% Asp), 10% by glutamate at A-site, 2% by lysine stretches and an additional 7% by negatively charged amino acids at the entrance to the exit tunnel.

### RNA structure and SNPs are not enriched at stall sites

mRNA structure has been shown to have an influence on translation slowdown (64,65). In bacteria, this pausing is presumed to be only transient, due to intrinsic helicase activity of the ribosome that can unwind thermodynamically stable mRNA structures (66,67) and predominantly unfolded state of mRNA inside cells (68). A recent study has also implicated its role in pausing of chloroplast ribosomes (15). Gawroński *et al.* observed increased stability of mRNA secondary structure 31 nucleotides downstream of the pausing site (MFE decreasing from -5.8 to -7.8 kcal/mol), for a sample of 78 stall sites. We investigated whether we could see any influence of structure in the CSSs that were not explained by sequence features. We analyzed *in silico* folded mRNAs, looking at the minimum free energy (MFE) around CSSs using the same method as previously. Our sample size was 627 CSSs, so significantly larger than in the chloroplast study; however, we were unable to find any decrease in MFE downstream of CSSs, and the overall MFE was < -10 kcal/mol (Supplementary Figure S6). This could be expected for strong ribosomal pauses due to the strong helicase activity of the ribosome and low mRNA folding levels in the cell. Naturally, the meta-analysis of multiple transcripts together does not capture individual structural features, and we can therefore not exclude that there could be structures contributing to stalling in individual cases. However, as a whole the group of transcripts do not exhibit increased structure downstream of CSSs leading to the conclusion that mRNA secondary structure is unlikely to be a major cause of conserved stall sites.

Synonymous single-nucleotide polymorphisms (SNPs) have been suggested to potentially induce stalling (69). While a synonymous SNP does not alter the amino acid sequence, it can change the codon to one that is rarely used. Such rare codons could provoke stalling (1,70) due to lower concentrations of the cognate tRNAs. To investigate this possible association, we searched de novo for SNPs as well as used the human SNP databases, containing over 36 million unique SNPs. We found no significantly different association of SNPs with stall sites as compared to random controls (see Materials and Methods section), indicating that stall sites are not generally associated with a higher incidence of SNPs.

### Conserved stall sites are enriched in genes related to RNA metabolism and co-translational protein folding

**Figure 3.**
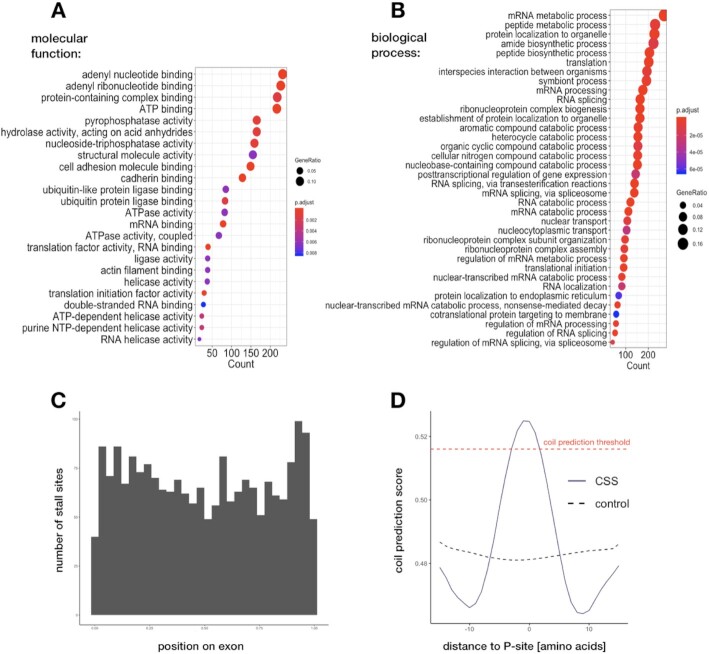
Functional characterization of CSS-containing genes. (**A** and **B**) Gene ontology enrichment analysis showing molecular functions and biological processes of conserved genes that are affected by stalling. (**C**) Position of CSSs on exons, with exon lengths normalized to 1. (**D**) Coil prediction score around CSSs, compared to control. Red dashed line marks the threshold typically used for coil prediction.

To investigate whether stalling plays a role in specific cellular processes, we sought to determine whether the genes containing CSSs share common functions. For instance, we observed a high degree of stalling conservation in ribosomal protein mRNAs, which are known to be translated slower than other transcripts with similar ribosome densities (71). Here, we found 119 CSSs in 88 ribosomal protein genes and mitochondrial ribosomal protein genes (87 CSSs and 71 genes in human libraries). To systematically explore enrichment, we compared our CSS-containing genes to a background of 4855 highly expressed genes using gene ontology analysis (Supplementary Table S6). This showed that 1212 CSS-containing genes (out of 1729) are associated with biological process ontology terms (Supplementary Table S4) and 766 with molecular function terms (Supplementary Table S7). The CSS-containing genes are enriched in functions related to RNA metabolic processes, mRNA splicing and processing, but also protein targeting to the endoplasmic reticulum (as in the case of *XBP1*), translation regulation, and co-translational protein targeting to the membrane. We also find terms related to nonsense-mediated decay which have been suggested as possible reasons for stalling (4,8,50,72,73) (see Figure 3B, Supplementary Figure S7, Tables S2 and S4). The results from GO analysis of subsets of CSSs, conserved in three or more organisms and for each organism separately were in line with these observations (Supplementary Figure S15), with the exception of fruit fly, which returned no GO enrichment. Similar analyses of control groups of well-expressed homologs: (i) with peaks, but without CSSs, and (ii) without any peaks compared to the same background returned no significant GO terms. To account for possible differences in ribosome coverage and translational efficiencies (Supplementary Figure S11A), we also constructed additional control sets: (iii) with similar ribosome coverage, but no CSSs and (iv) with similar translational efficiency, but no CSSs (see Supplementary Figure S11B,C and Materials and Methods section). These too did not return any terms related to RNA processing. Finally, to account for minor differences in alignment scores and peak numbers between CSSs and the genes with peaks but without CSS (control group 1), we selected subsets of the latter (v) with similar alignment score and (vi) similar peak number (see Supplementary Figure S11D,E, and Materials and Methods section). We found no GO enrichment in these sets. Together, this argues that stalling serves a specific function in the cell regulating a subset of genes involved in RNA metabolism and protein targeting.

The enrichment of various ’binding’ terms and enzymatic activities in the molecular function ontology (Figure 3A) could be related to the overrepresentation of Pro/Gly/Glu/Asp amino acids around CSSs. These amino acids tend to be located within disordered regions that in general facilitate binding and folding (74,75). Proline contributes to conformational rigidity of a protein, while glycine provides flexibility. Both are often found within turn and loop structural regions and play an important role during chain compaction early in folding (76–78). Glutamic acid is the second most disorder-promoting amino acid (79,80). Being positioned within or in the close proximity to the enzyme catalytic sites, the two acids, Glu and Asp, play important roles in enzyme active centers (80), while Gly and Pro are responsible for the architectonics of the active site (81).

To test the possibility of whether conserved stall sites could be involved in co-translational folding, we first looked at positioning of CSSs in exons. When comparing the positioning of human CSSs relative to the closest splice site we found that stall sites tend to be preferentially positioned closer to the 3’end of exons (Figure 3C). As secondary structure elements tend to be contained within exons (82), stall sites at the ends of exons might be there to allow the newly synthesized regions to order themselves, as suggested previously (83–85). We therefore predicted the level of disorder in the CSS-containing proteins using DisEMBL (47) (see Materials and Methods section). This analysis revealed that CSSs tend to be located within coils, which are the linkers between alpha-helices and beta-strands (Figure 3D) as often as in 65% cases (with the average 55% for the random sites sampled from the same proteins, *P*-value < 2.2e-16). To see if similar dependence exists for tertiary structure elements, we analyzed distance to the closest upstream protein domain downloaded from the CATH database. However, we found no evidence that CSSs are more likely located downstream of higher-order domains than random (Supplementary Figure S8). Overall, a subset of CSSs might be involved in the co-translational folding of secondary structure protein domains.

### Conserved stall sites are not associated with membrane targeting

Stalling has been shown to facilitate co-translational protein targeting to the membrane (5,86). In a recent study, a significant pausing signal was observed downstream of the start of transmembrane domains (TMs) in chloroplasts. It occurred 52 amino acids downstream of the start of type II TMs and 34 amino acids for type I TMs (15). The authors speculated that this would leave the time for the TMs to fold before translation would proceed. To investigate whether this type of stalling is a conserved mechanism, we downloaded all 1512 TM type I and 464 type II proteins available in UniProt for human. Out of these, only 76 and 36 contained CSSs, respectively. The CSSs that were present in the TM proteins were distributed randomly over the body of the gene, and not over-represented at any specific position downstream of the start of TMs. Therefore, we conclude that our conserved stall sites are not associated with folding of transmembrane domains.

### Most conserved stall sites do not lead to aborted translation

Some stall sites might be a consequence of aberrant translation. To investigate this hypothesis we sought to understand whether stalling could lead to abortion of translation. We therefore calculated the ratio of mean ribosome coverage upstream versus downstream of all CSSs in the four human libraries. We found that for the majority of stall sites the ratio is <2-fold, and only 22 have log2 ratio higher than 2 (see Supplementary Figure S9). From manual inspection of these we found 13 stall sites in 11 genes that might lead to programmed abortion of translation, possibly by NMD or NGD (Supplementary Table S5), as these were located over out-of-frame stop codons or just before alternative exons.

Similarly, knowing that canonical termination of translation produces longer footprints due to conformational change of the ribosome (16), we investigated whether we could find longer footprints at CSSs if these led to termination or different fragment length distribution whatsoever. Indeed, we observe a shift in average footprint length around stop codons in the four human libraries (Supplementary Figure S10A). However, no such shift is observed around CSSs present in these libraries (Supplementary Figure S10B). Overall, we conclude that CSSs do not tend to lead to abortion of translation or conformational change of the ribosome.

## CONCLUSION

The aim of this study was to identify and characterize conserved functional stall sites. Using a representative set of libraries with good coverage for five model organisms, we tailored processing to each dataset separately to allow for robust comparison. We identified thousands of library-specific peaks, of which 3293 stall sites were conserved in at least two organisms.

While many mechanisms have been suggested to induce stalling we found that 63% of our conserved stall sites can be explained by proline, glycine and negatively charged amino acids. Importantly, this does not exclude that RNA structure, positively charged amino acids or other previously reported features contribute to stalling across the organisms studied here, nor does it suggest that these mechanisms are not conserved. However, for stall sites that can be recovered in the same position in the same genes across these species these other features do not seem to play a significant part.

Interestingly, many of the CSSs identified in this study are present in genes coding for RNA processing factors, notably splicing factors. While this can partly be a consequence of which genes in general are conserved across species, these genes are specifically enriched relative to other conserved genes indicating a functional significance. Whether this function is related to unconventional cytoplasmic splicing (as is the case with XBP1) is however unclear. Another interesting category are genes with functions related to co-translational folding, which are thought to be regulated by stalling (2–4). We find that CSSs tend to be located between ordered protein domains, suggesting that some could play a role in co-translational folding. However, as proteins with CSSs are involved in co-translational processes themselves, this might imply a possible self-regulation mechanism, where stalling regulates the synthesis of such proteins, but in turn, the synthesized proteins regulate the stalling during translation. This is an attractive hypothesis for which our study provides candidates for further experimental testing.

Given the high variability in terms of cell lines and tissues of the data analyzed here, it is likely that the conserved stalling landscape is substantially larger than the sites identified in this study and includes stall sites that are specific to certain conditions. Indeed the subset of CSSs identified here may be predominantly stall sites occurring in conserved genes that are active across most cell types, e.g. house-keeping genes. Beyond these, there are also likely numerous non-conserved, organism- and/or condition-specific stall sites, but these are outside the scope of this study. Also, although deeply conserved at its core, the ribosome has been demonstrated to show variation in its components (87,88). This could have an impact on regulation of translation, and potentially influence heterogeneity of stalling triggers. This is an interesting avenue for future research. Finally, the vast majority of ribosome profiling experiments perform size-selection, keeping only the footprint sizes of 28–29 nucleotides, typical of the non-rotated, elongating ribosomes, but it has been shown that lengths one might expect at stall sites include those from closely stacked di-ribosomes protecting around 80 nucleotides (89) or short footprints 20–22 nucleotides long representing the rotated form of the ribosome (90). Therefore, keeping longer and shorter fragments might be beneficial for analysis of stalling.

In conclusion, this study presents a rich resource on global, conserved ribosome stall sites, indicating possible causes and implications. The methods and data presented here lay the foundation for further research involving in-depth molecular biology to characterize the functional relevance of the identified conserved stall sites.

## DATA AVAILABILITY

All the scripts used for the analysis in this work are available at https://github.com/katchyz/stalling. Intermediate data files are available via Zenodo at https://zenodo.org/record/4589132.

## Supplementary Material

lqab038_Supplemental_Files
