# Supplementary material for: Deep conservation of ribosome stall sites across RNA processing genes
Source: NAR Genom Bioinform. 2021 May 25;3(2):lqab038. doi: 10.1093/nargab/lqab038 (PMC8152447; doi:10.1093/nargab/lqab038)
Supplement: lqab038_Supplemental_Files [file lqab038_supplemental_files.zip › stalling_Supp.pdf]

Supplementary material: Deep conservation of ribosome stall sites across RNA processing genes

The supplementary tables 2-7 are available in the XLSX file.

Supplementary Table 1: Data overview.

Supplementary Table 2: Peak statistics.

Supplementary Table 3: Conserved stall sites.

Supplementary Table 4: Gene Ontology biological processes and associated genes with CSSs.

Supplementary Table 5: Genes and stall sites that might lead to translation degradation.

Supplementary Table 6: Peak statistics on all well-expressed homologs.

Supplementary Table 7: Gene Ontology molecular functions and associated genes with CSSs.

Supplementary table 1: Data overview. (*) Treatment with cycloheximide was performed *after* flash-freezing the embryos.

Library name	Organism	Strain /cell line / stage	SRA accession	Translation inhibitor	Footprint lengths and offsets																					Reference
					20	21	22	23	24	25	26	27	28	29	30	31	32	33	34	35	36	37	38	39	40	
Y1	yeast	gb15	SRR387905	CHX		12	12	12	12	12	12	12	12	12	12	12	12	12	12							Brar *et al.* 2012
Y2		BY4741	SRR1520317	CHX					8	9	10	11	12	13	14											Gerashchenko *et al.* 2014
Y3			SRR1520325	no drug						9	10	11	12	13	14	14	14									
F1	fruit fly	S2 cells	SRR942879	emetine							12	12	12	12	12	13	14	14	14	14	14	14	14	14	15	Dunn *et al.* 2013
F2			SRR6930625	DMSO			8	8	8	11	11	12	12	13	13	13	14	14	14							Luo *et al.* 2018
F3			SRR3031135	emetine		5	5	5	6	7	9	9	10	11	12	13	13	13	13	13						
Z1	zebrafish	embryo, 2hpf	SRX399824, SRX399826, SRX399828	CHX*	3	4	5	6	7	8	9	10	12	12	12	13										Bazzini *et al.* 2014
Z2			SRR5893147, SRR5893148	CHX*						9	10	11	12	12	13	14										Beaudoin *et al.* 2018
Z3			SRR1039873	CHX							12	12					12									Subtelny *et al.* 2014
Z4		Embryo, 4hpf	SRR836195	CHX*						8	9	11	11	12	12	13										Chew *et al.* 2013
Z5			SRR1039876	CHX											12											Subtelny *et al.* 2014
Z6		embryo, 6hpf	SRR836196	CHX*						8	8	11	11	12	12	13	13									Chew *et al.* 2013
Z7			SRR1039879	CHX											12											Subtelny *et al.* 2014
M1	mouse	ESC	SRR315601, SRR315602	CHX		3	3					12	12	12	12	13	14	14	14	14						Ingolia *et al.* 2011
M2			SRR315616, SRR315617, SRR315618, SRR315619	no drug						7	8	9	10	11	12	13	14	14	14	14					14	
M3		3T3	SRR1039863	CHX					11	11	11	12	13	13	13	13	13	13	13	13					14	Subtelny *et al.* 2014
H1	human	fibroblasts	SRR609197	CHX			5	5	5	8	8	8	11	12	12	13	13	14	14	14	14				14	Stern *et al.* 2012
H2			SRR592961	no drug		3	4	5	6	7	8	9	10	11	12	13	14	14	14	14					14	
H3		HeLa	SRR970587	CHX	3	4	5	6	6	7	8	9	12	12	12	13	13	14	14	14						Stumpf *et al.* 2013
H4		HEK293	SRR1039861	CHX						13	13	13	13	13	13	13	13	13	13	13						Subtelny *et al.* 2014

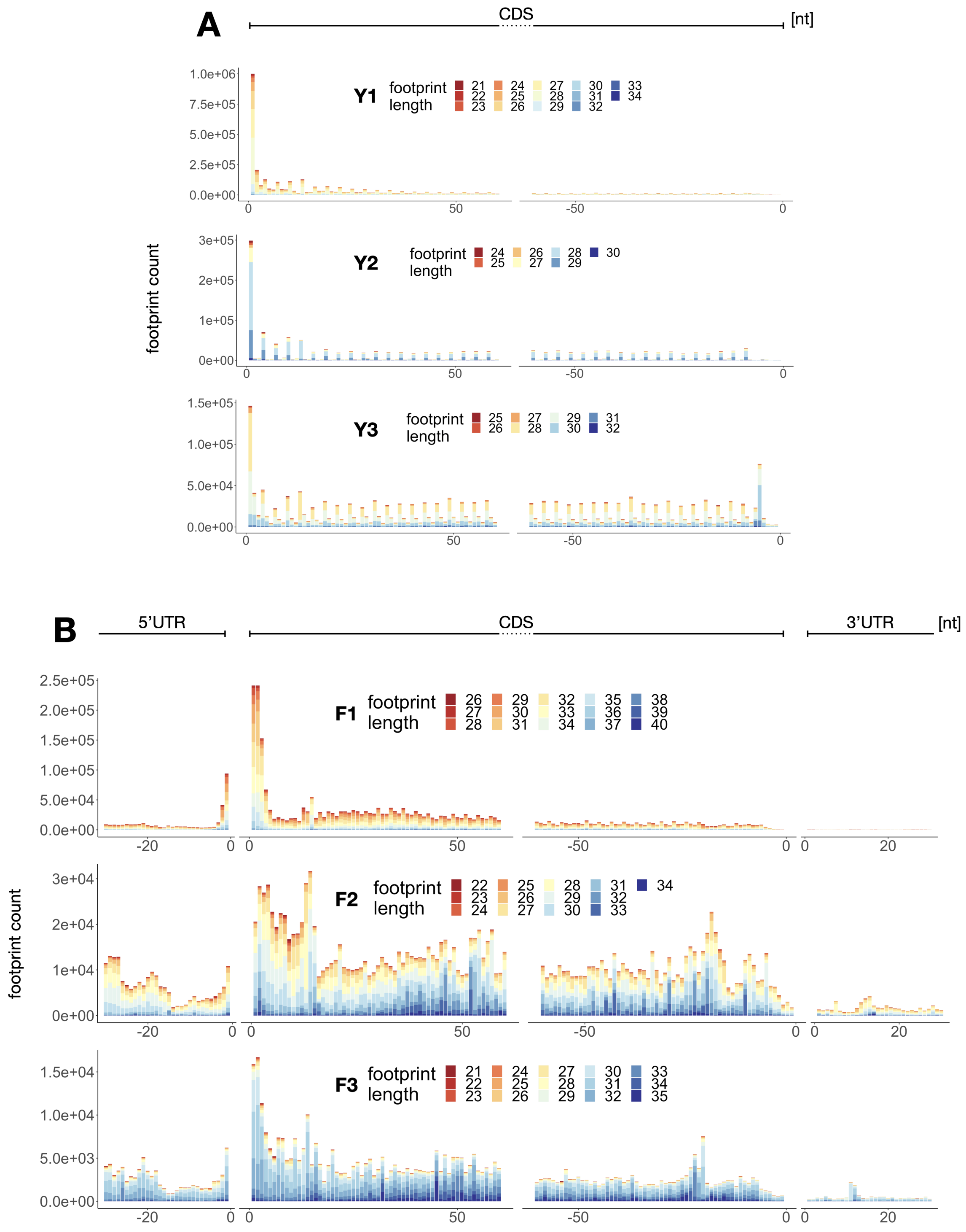
A
CDS
[nt]
Y1
footprint length
21 22 23 24 25 26 27 28 29 30 31 32 33 34
1.0e+06
7.5e+05
5.0e+05
2.5e+05
0.0e+00
0
50
-50
0
Y2
footprint length
24 25 26 27 28 29 30
3e+05
2e+05
1e+05
0e+00
Y3
footprint length
25 26 27 28 29 30 31 32
1.5e+05
1.0e+05
5.0e+04
0.0e+00
footprint count
B
5'UTR
CDS
3'UTR
[nt]
F1
footprint length
26 27 28 29 30 31 32 33 34 35 36 37 38 39 40
2.5e+05
2.0e+05
1.5e+05
1.0e+05
5.0e+04
0.0e+00
-20
0
50
-50
20
F2
footprint length
22 23 24 25 26 27 28 29 30 31 32 33 34
3e+04
2e+04
1e+04
0e+00
F3
footprint length
21 22 23 24 25 26 27 28 29 30 31 32 33 34 35
1.5e+04
1.0e+04
5.0e+03
0.0e+00
footprint count

Supplementary Figure 1: Ribosome meta-profiles of reads assigned to P-site for each library. (**A**) yeast, (**B**) fruit fly, (**C**) zebrafish, (**D**) mouse and (**E**) human. On x-axis: last 30 bases of 5′UTR, first and last 60 bases of CDS and first 30 bases of 3′UTR. Ribosome footprints are separated by length, ranging from short (red) to long (blue) fragments. The peaks visible over start codon and the last sense codon (positions 0 to 2 and -5 to -3 on CDS), clear periodicity and high coverage within CDS compared to UTRs indicate proper assignment of reads to P-site. Peaks at start codon and increased coverage on the first few codons of CDSs are clear in CHX and other translation inhibitors-treated libraries (Y1, Y2, F1, F2, F3, Z3, Z5, Z7, M1, M3, H1, H3, H4), while the peak at translation stop is visible in flash-frozen libraries (Y3, Z1, Z2, Z4, Z6, M2, H2).

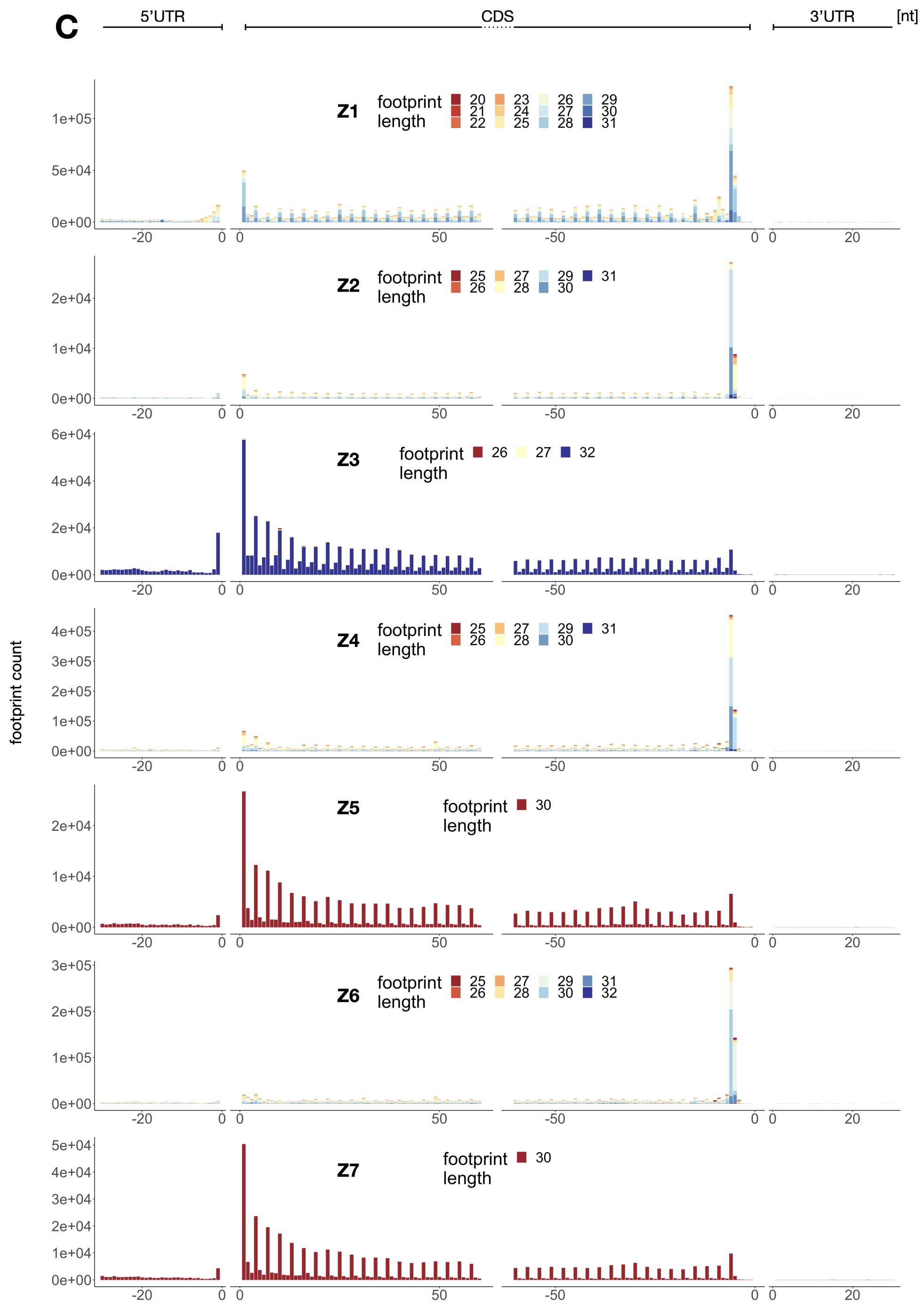

Supplementary Figure 1: - continuation.

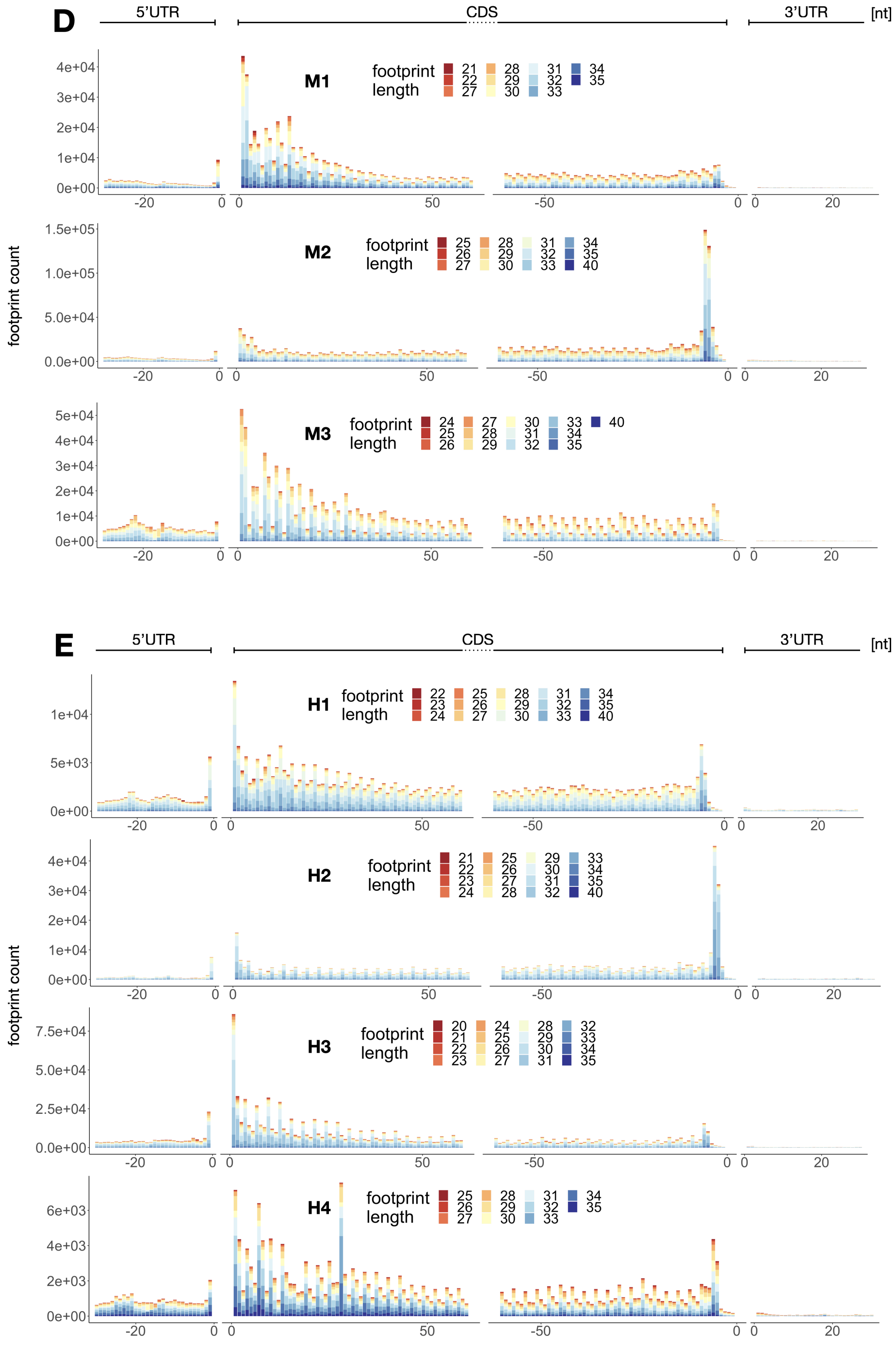

Supplementary Figure 1: - continuation.

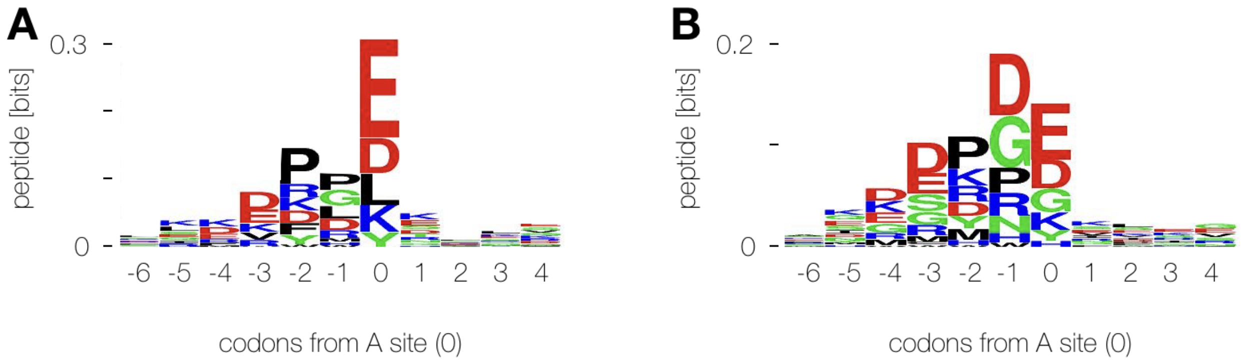

Supplementary Figure 2: Peptide motif around stall sites. (**A**) Motif in M2 library, as reported previously in [29] and (**B**) consensus peaks from M1 and M2 libraries.

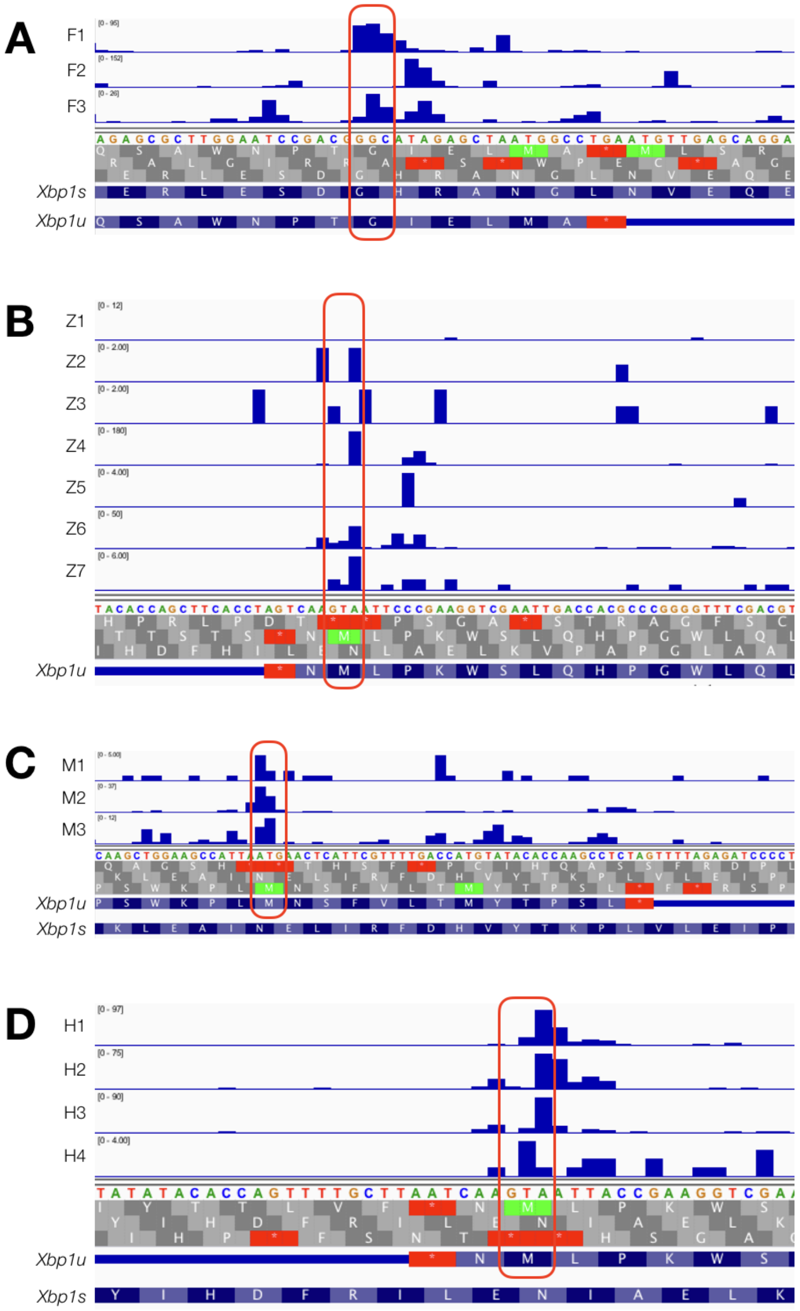

Supplementary Figure 3: *Xbp1* stall site. (**A**) Ribosome coverage in fruit fly, (**B**) zebrafish, (**C**) mouse and (**D**) human libraries.

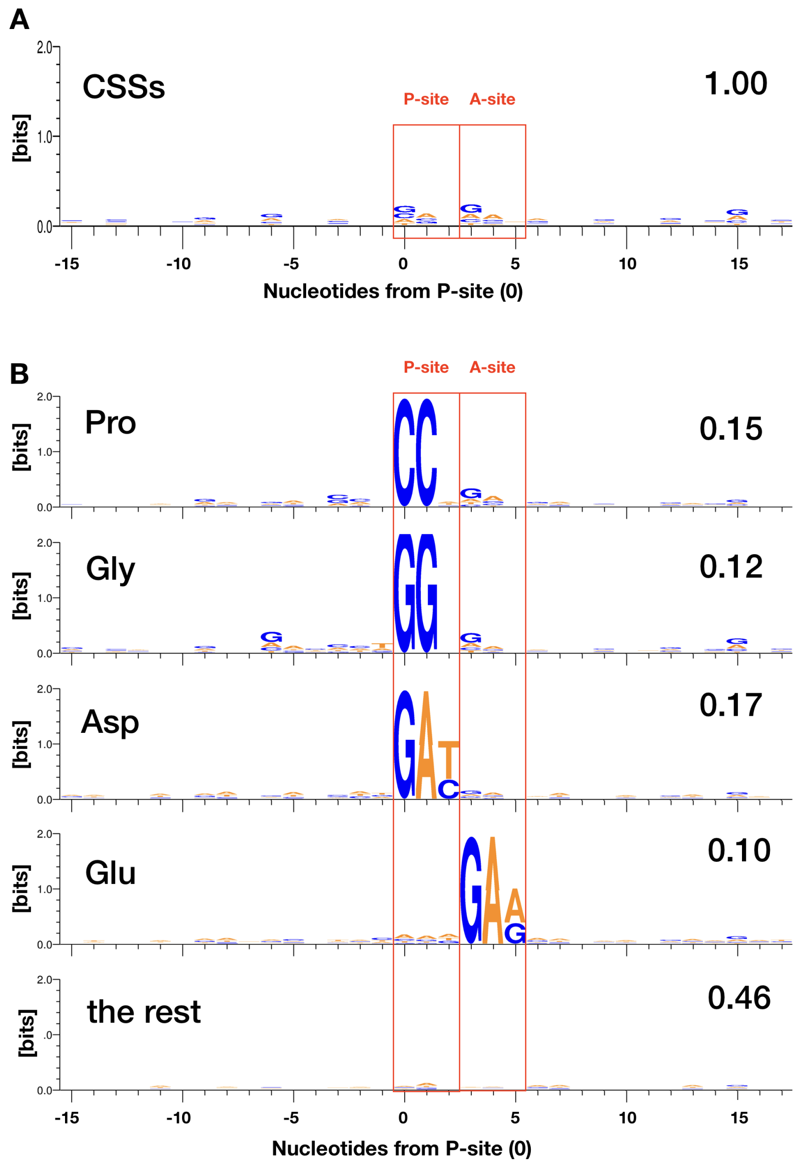

Supplementary Figure 4: Nucleotide bias around conserved stall sites. (**A**) Sequence logo for CSSs. (**B**) Sequence logos split by the most significant contributors: prolines, glycines, aspartate at P-site and glutamate at A-site, accounting for 54% CSSs together, and logo of the remaining 46% sequences. The amino acids show biased context, which is not visible on control logos for all of these amino acids extracted from the same transcripts (not shown).

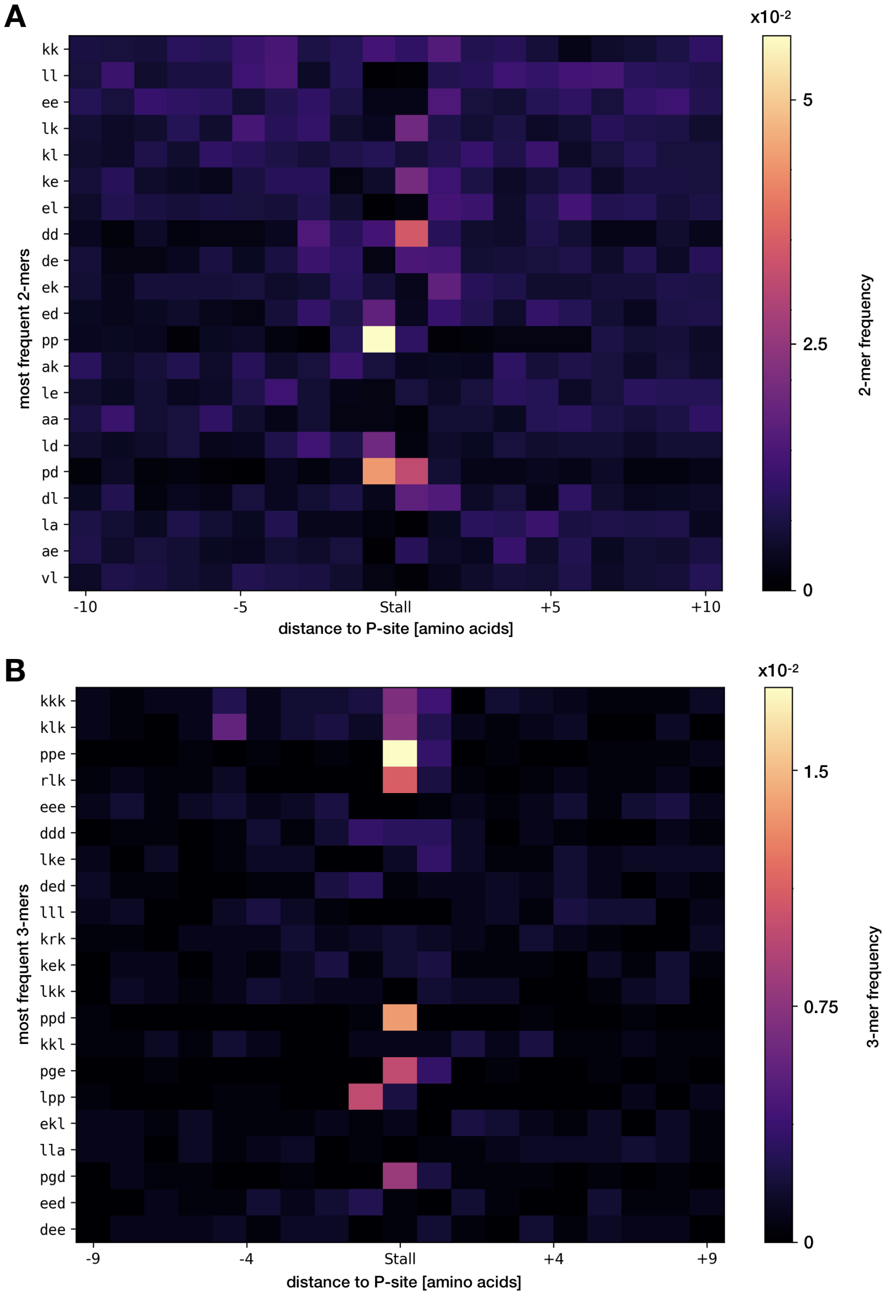

Supplementary Figure 5: Overrepresentation of amino acid k-mers around stall sites. (**A**) 2-mers and (**B**) 3-mers, sorted by frequency of occurrence in CSSs, top to bottom.

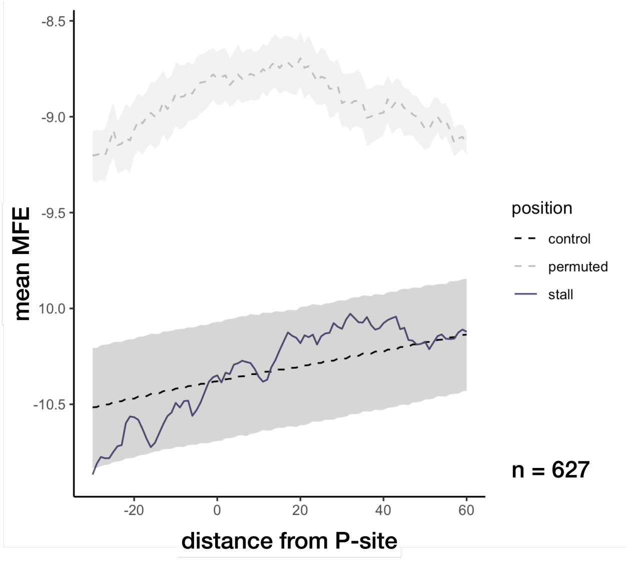

Supplementary Figure 6: Structure around stall sites. Mean minimum free energy of regions around CSSs that could not be explained by sequence features (CSS, purple solid line), compared to background of random sites on the same transcripts (control, black dashed line) and the same regions around CSSs but with permuted codons (permuted, gray dashed line). The shaded regions represent areas between the 5th and 95th quantile of MFE distributions for sampled data (control and permuted).

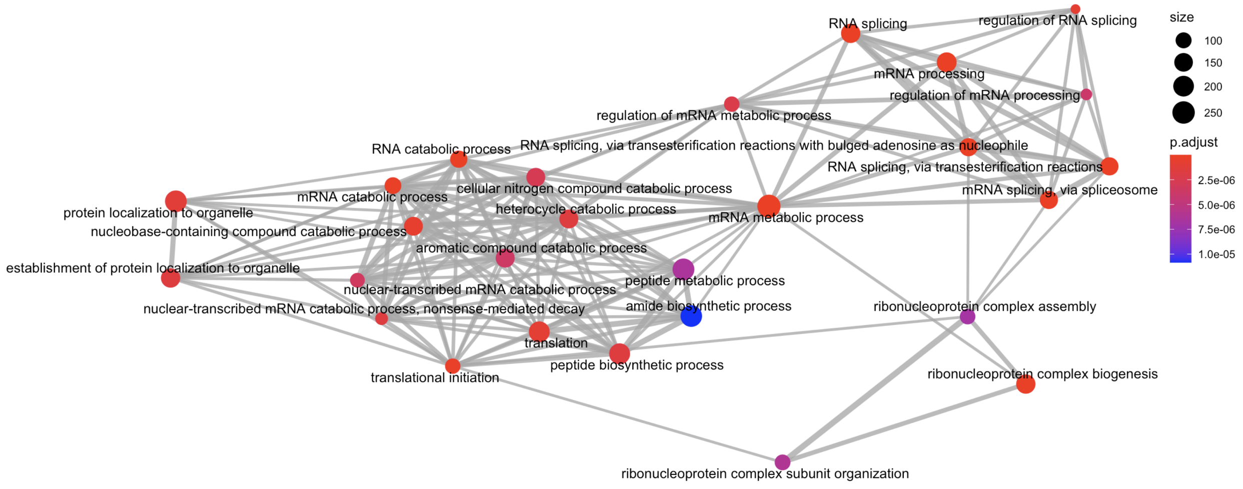

Supplementary Figure 7: Gene ontology enrichment map. Enrichment for over-representation of biological processes, as in Figure 3B.

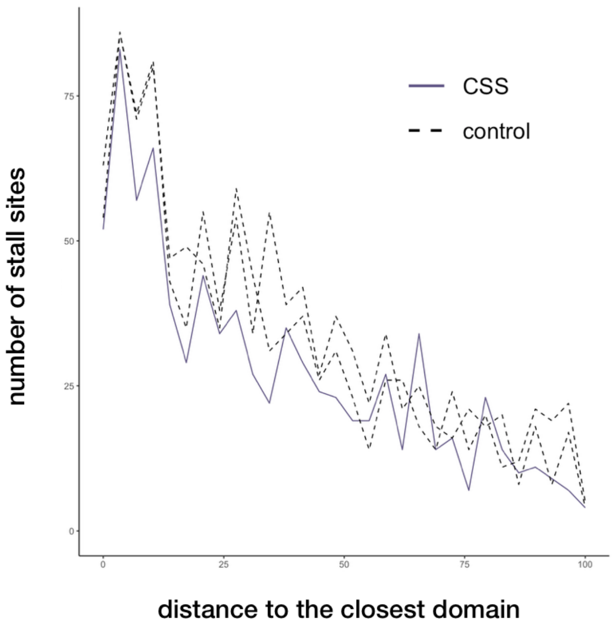

Supplementary Figure 8: Distance to transmembrane domains. Distance of the CSSs to the end of the closest upstream protein domain, compared to random controls.

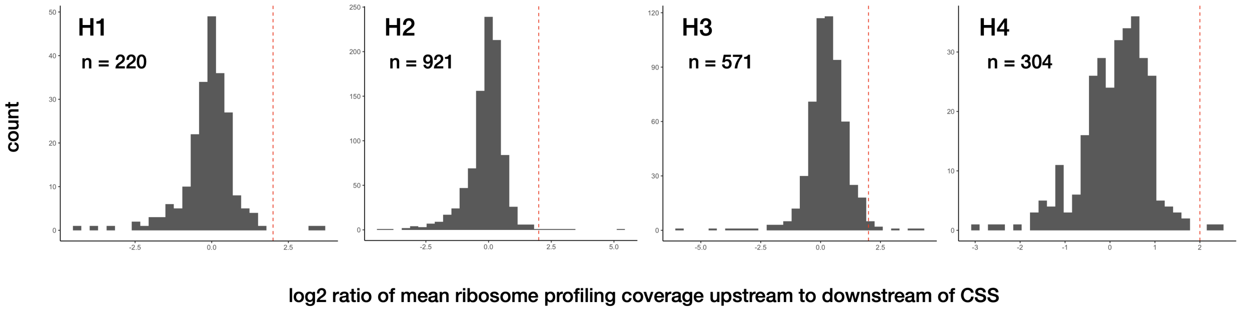

Supplementary Figure 9: Stalling and degradation. Change fold of the ribosome profiling coverage upstream of the CSS to downstream for the four human libraries. Red dotted line is set at log2 ratio = 2, with transcripts to the right being possible candidates for stalling-regulated mRNA degradation.

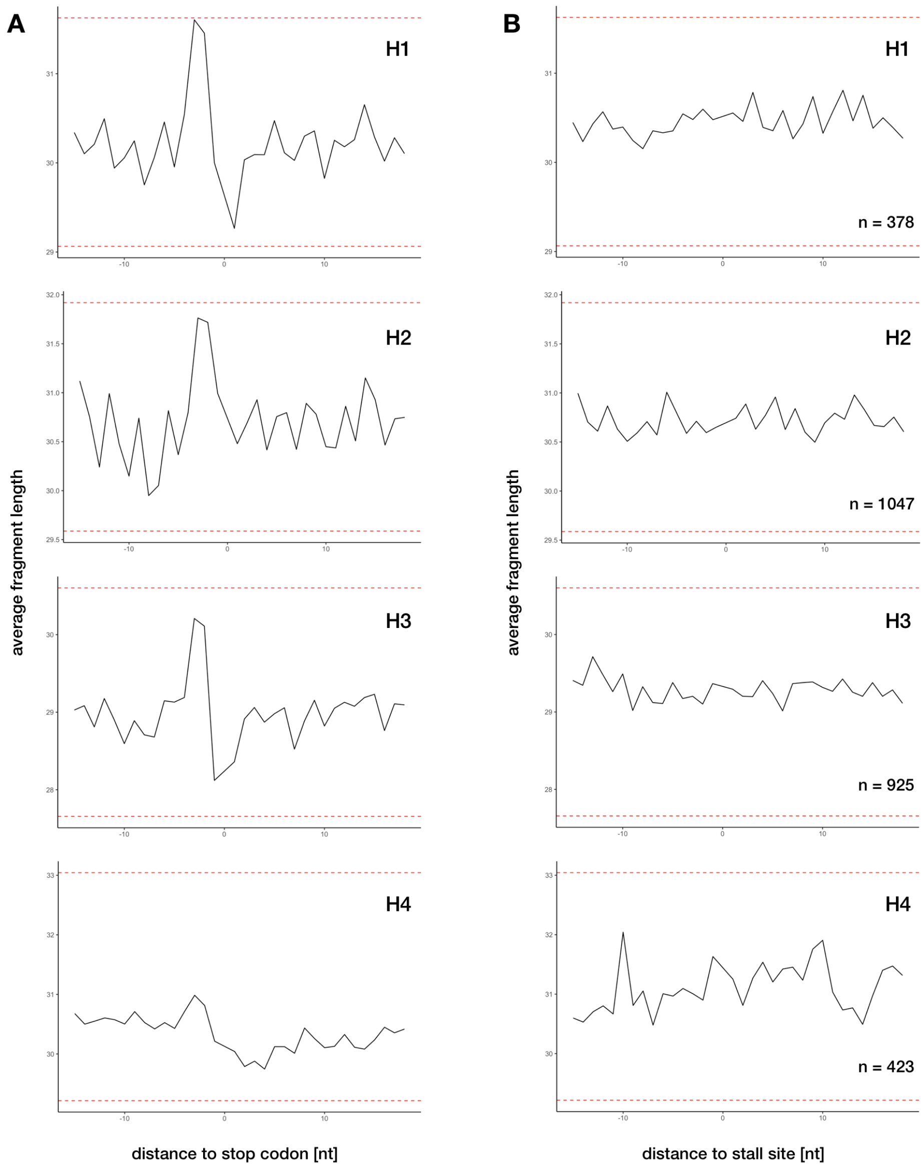

Supplementary Figure 10: Shift in fragment length distribution. Average fragment length distribution around (**A**) stop codons and (**B**) CSSs present in four human libraries, H1-H4. The red lines indicate 10% tails of the fragment length distribution calculated from the whole libraries.

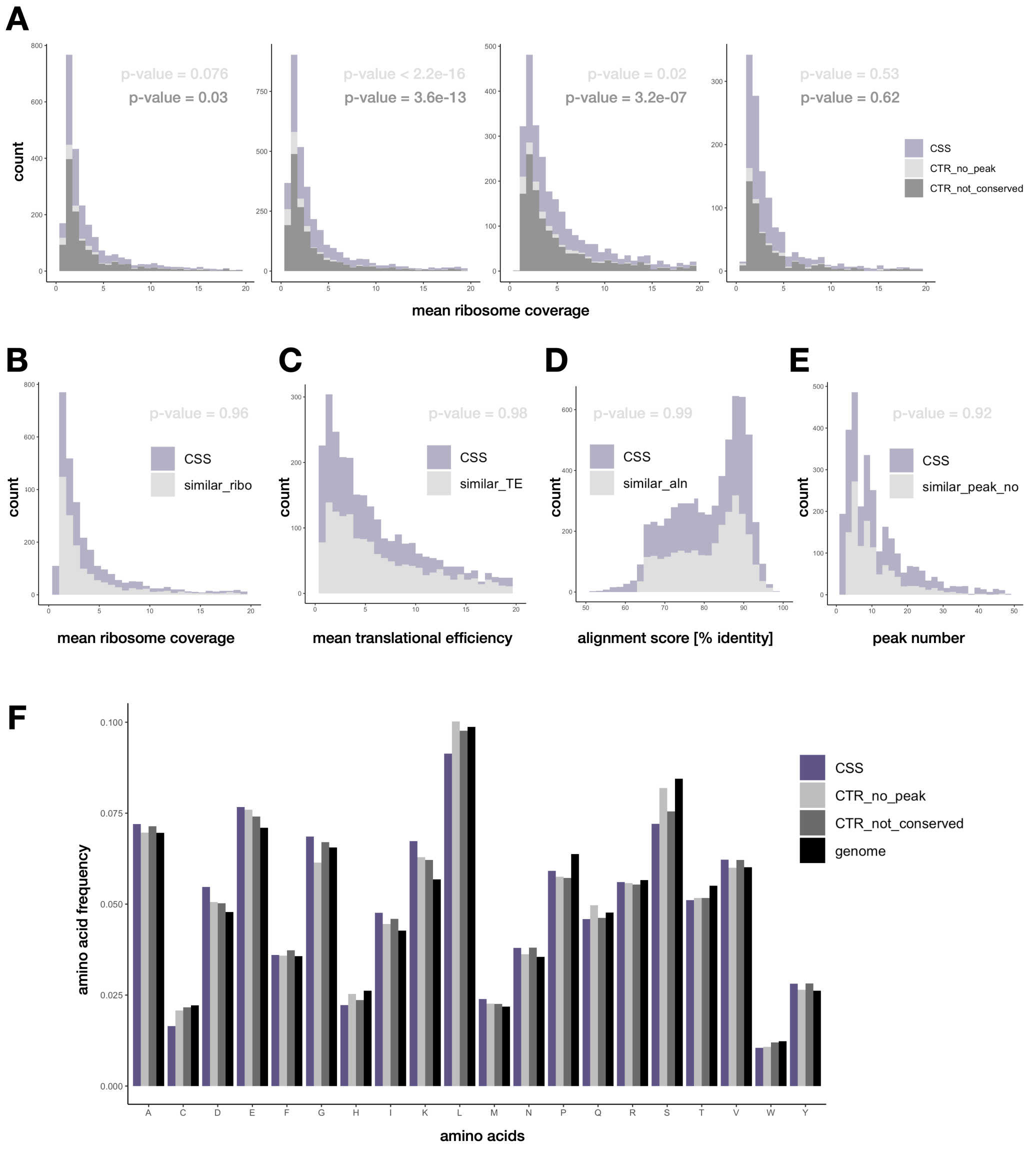
A
p-value = 0.076
p-value = 0.03
p-value < 2.2e-16
p-value = 3.6e-13
p-value = 0.02
p-value = 3.2e-07
p-value = 0.53
p-value = 0.62
CSS
CTR_no_peak
CTR_not_conserved
count
mean ribosome coverage
B
p-value = 0.96
CSS
similar_ribo
count
mean ribosome coverage
C
p-value = 0.98
CSS
similar_TE
count
mean translational efficiency
D
p-value = 0.99
CSS
similar_aln
count
alignment score [% identity]
E
p-value = 0.92
CSS
similar_peak_no
count
peak number
F
CSS
CTR_no_peak
CTR_not_conserved
genome
amino acid frequency
A C D E F G H I K L M N P Q R S T V W Y
amino acids

Supplementary Figure 11: Ribosome coverage, translational efficiency, and amino acid frequency distributions. (**A**) Distributions of mean ribosome coverage for transcripts in the four human libraries. CSS-containing genes are shown in purple and controls in gray. P-values for differences in distributions resulting from Mann-Whitney U tests for CSS versus each control are shown in the colours corresponding to those controls. (**B**) Distributions of mean ribosome coverage for CSS-containing genes vs control of similar ribosome coverage. (**C**) Distributions of translational efficiencies for CSS-containing genes vs control of similar TE. (**D**) Distributions of homology alignment scores for CSS-containing genes vs control of similar scores. (**E**) Distributions of peak number in CSS-containing genes vs control of similar numbers. (**F**) Distributions of amino acids for CSS-containing genes and two controls. Shown in black are amino acid distributions for all human proteins.

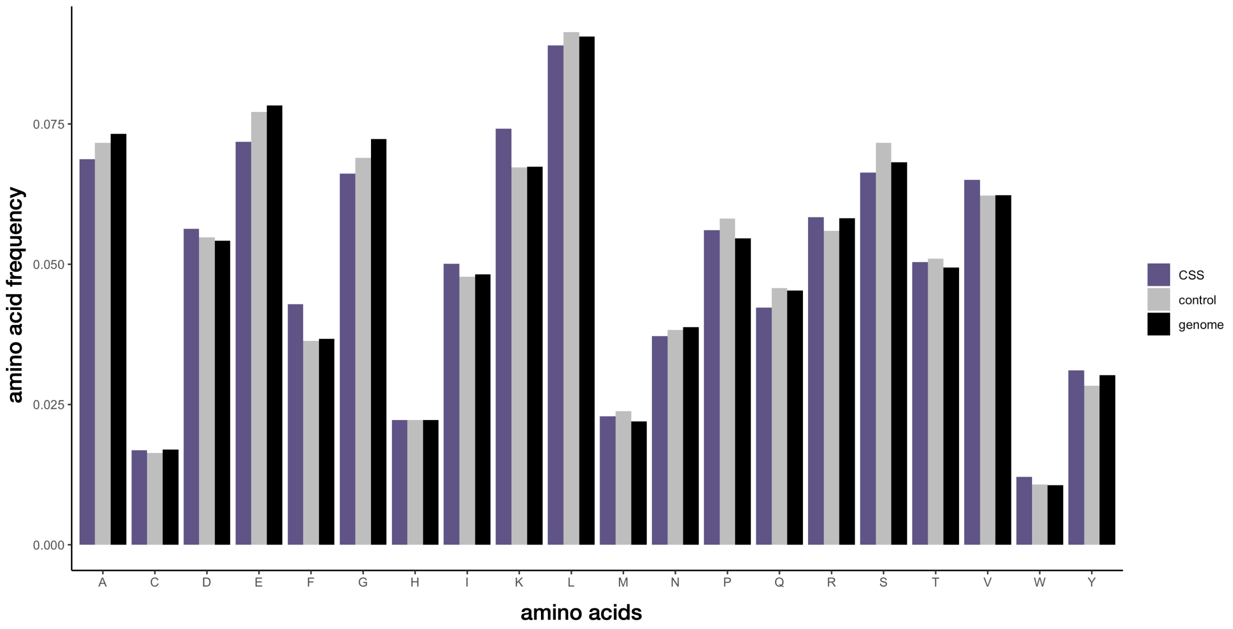

Supplementary Figure 12: Nascent peptide composition. Distributions of amino acid frequencies in the 40 amino acids upstream of CSSs (purple) and random control sites (gray), spanning the ribosome exit tunnel. Shown in black are amino acid distributions of all human proteins.

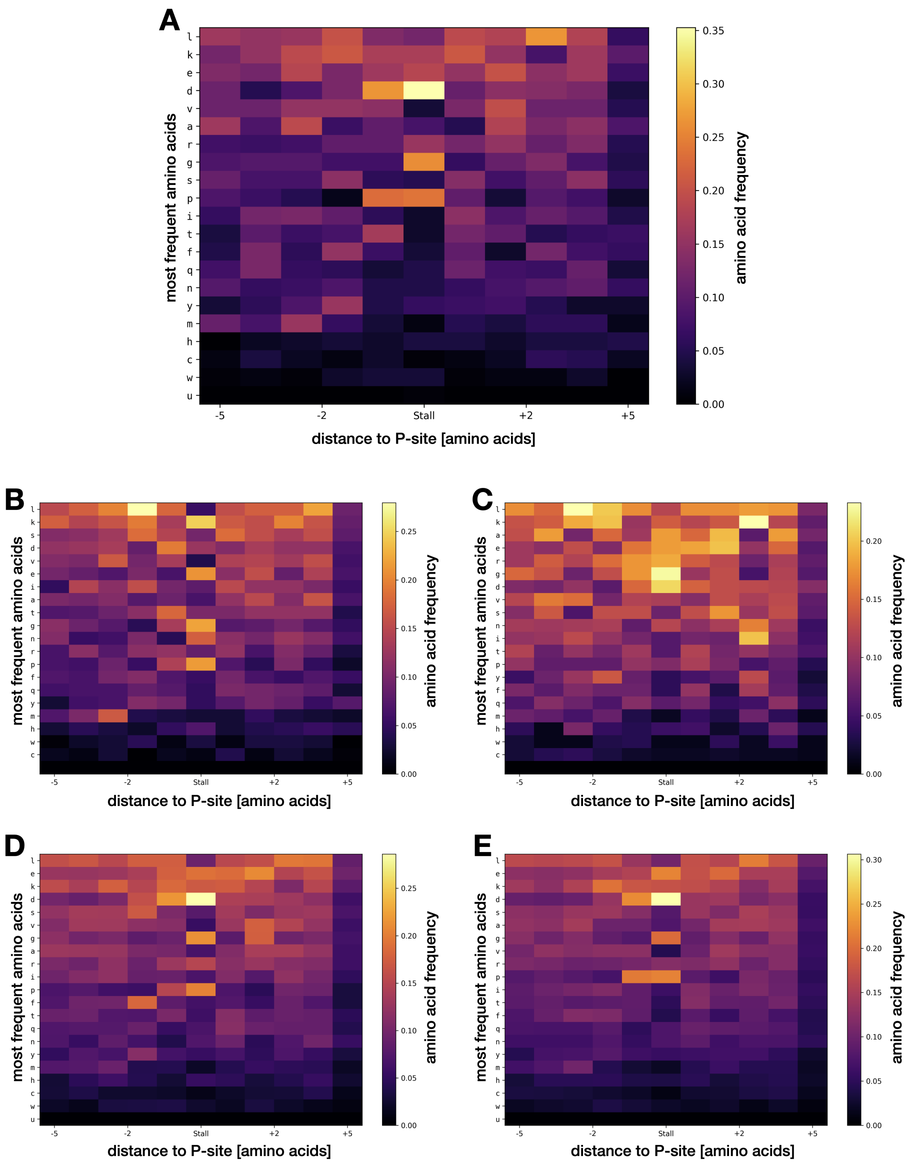

Supplementary Figure 13: Amino acids around subsets of CSSs. Overrepresentation of amino acids around CSSs, sorted from most to least frequent, top to bottom, for (**A**) CSSs conserved in at least 3 organisms (human amino acid sequences), (**B**) yeast, (**C**) fruit fly, (**D**) zebrafish and (**E**) mouse. In B-E the CSSs are conserved in the given organism and at least one other.

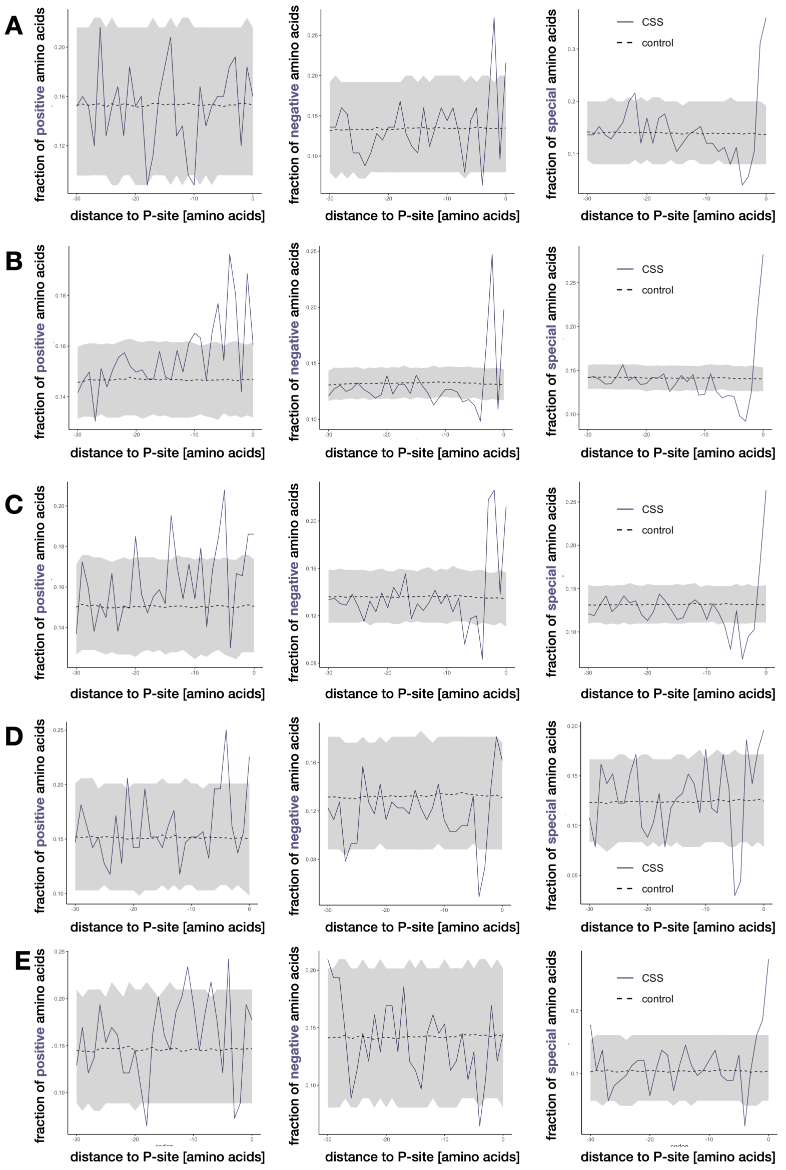

Supplementary Figure 14: Amino acid charge around subsets of CSSs Fraction of positively charged amino acids in the 30 amino acids upstream of the CSSs at P-site (0), spanning the exit tunnel versus background (shaded regions show background distribution, from 5th to 95th percentile) for (**A**) CSSs conserved in at least 3 organisms (human sequences), (**B**) mouse, (**C**) zebrafish, (**D**) fruit fly, and (**E**) yeast. In B-E the CSSs are conserved in the given organism and at least one other.

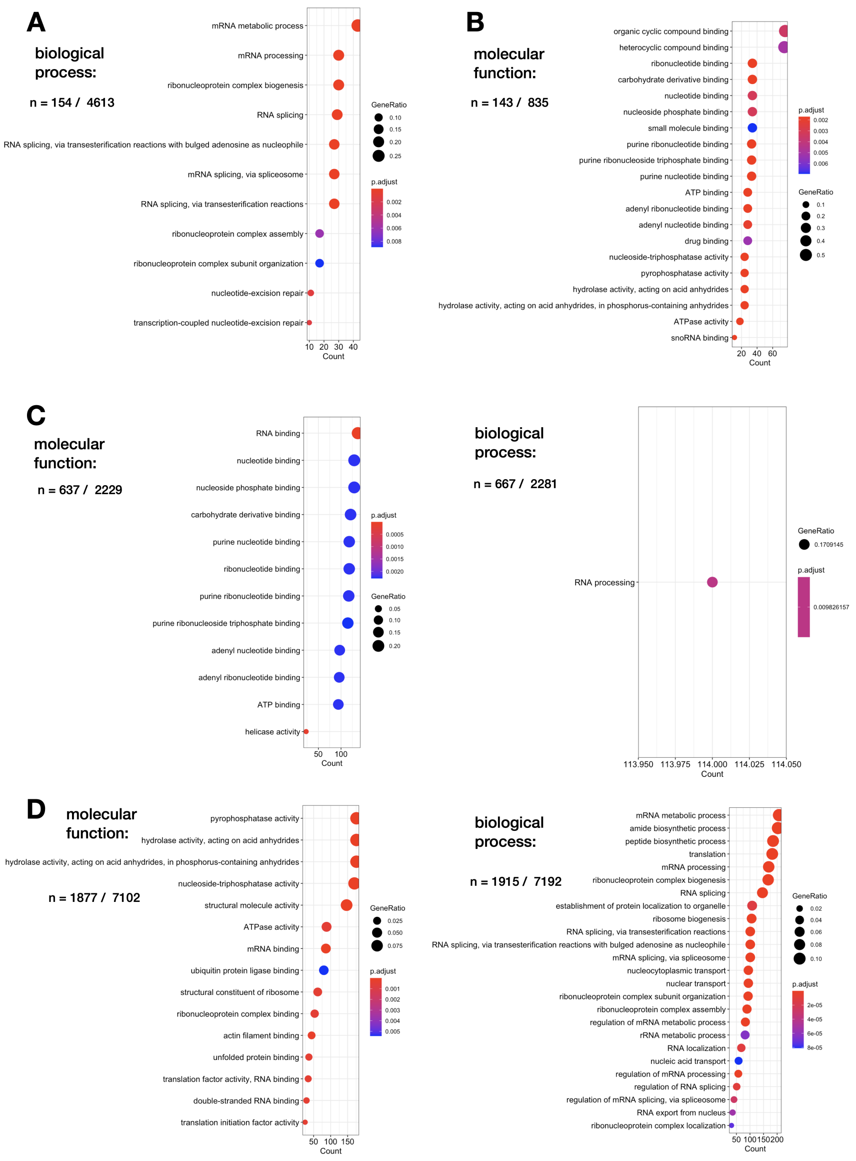

Supplementary Figure 15: GO for subsets of CSS-containing genes. Gene ontology enrichment for CSS-containing genes, where (**A**) CSS is conserved in at least 3 organisms, (**B**) yeast, (**C**) zebrafish, and (**D**) mouse. In B-D the CSSs are conserved in the given organism and at least one other. Shown also are the numbers of genes in the sample versus in the background.
